# Acridinium Chemiluminogenic Labels—Synthesis, Analytical Performance, and Mechanism of Light Generation—A Comparison in View of Biomedical Diagnostics

**DOI:** 10.3390/molecules31061041

**Published:** 2026-03-20

**Authors:** Karol Krzymiński, Beata Zadykowicz, Justyna Czechowska, Paweł Rudnicki-Velasquez, Illia Serdiuk, Adam K. Sieradzan, Lucyna Holec-Gąsior

**Affiliations:** 1 Faculty of Chemistry, University of Gdańsk, 80-308 Gdańsk, Poland; adam.sieradzan@ug.edu.pl; 2 Cyprotex Discovery Ltd., No. 24 Mereside, Alderley Park, Nether Alderley, Cheshire SK10 4TG, UK; Justyna.Czechowska-Kryszk@cyprotex.com; 3 Department of Falsified Medicines and Medical Devices, National Medicines Institute, Chełmska 30/34, 00-725 Warsaw, Poland; p.rudnicki@nil.gov.pl; 4 Faculty of Mathematics, Physics and Informatics, University of Gdańsk, Wita Stwosza 57, 80-308 Gdańsk, Poland; illia.serdiuk@ug.edu.pl; 5 Department of Molecular Biotechnology and Microbiology, Faculty of Chemistry, Gdańsk University of Technology, Narutowicza 11/12 Str., 80-233 Gdańsk, Poland; lucholec@pg.edu.pl

**Keywords:** acridinium chemiluminescence, labelling, immunodiagnostics, statistics, DFT calculation, semi-empirical approach, surfactants

## Abstract

This paper presents the synthesis, physicochemical characterisation, and analytical applications of chemiluminescent (CL) labels based on acridinium salts (ALs) for biomedical diagnostics. These compounds emit light as a result of oxidative reactions and represent an established class of reagents widely employed in chemiluminescence immunochemical assays (CLIAs) today. A series of structurally differentiated acridinium labels (**AL1**–**AL5**) was synthesised applying mostly original synthetic routes and purified to chromatographic purity (>90%, RP-HPLC). The compounds, including a commercial product treated as a reference, were successfully conjugated to anti-human IgG, yielding stable immunochemical reagents suitable for immunoassays with CL detection. The chemiluminescence properties of the obtained labels and their protein conjugates were investigated in aqueous buffers and in the presence of surfactants. The emission profiles exhibited characteristic flash-type kinetics with emission maxima occurring within 0.15–0.25 s after reaction initiation. The presence of surfactants more or less significantly enhanced the emission intensity, with signal increases of up to approx. 2-fold compared to surfactant-free systems. Analytical calibration demonstrated a linear response of signal derived from native labels over at least one order of magnitude of concentration, with detection limits falling in the range of 10^−9^–10^−10^ M, confirming the high sensitivity of the developed compounds. The experimental results were supported by theoretical studies using density functional theory (DFT), which confirmed the energetic feasibility of the CL reaction pathway and identified structural factors influencing activation barriers. Additional semiempirical calculations (PM7) indicated that the dielectric environment and proximity of ionic species can influence the reaction energetics, providing mechanistic support for the experimentally observed effects of surfactants. The results demonstrate that both molecular structure and microenvironment influence CL efficiency and kinetics of the investigated systems. The developed acridinium labels exhibit analytical performance better or comparable to commercial reagents and are fully compatible with standard immunodiagnostic conjugation protocols, confirming their suitability for use in modern chemiluminescent immunoassays.

## 1. Introduction

Chemiluminescence-based detection is widely employed in modern bioanalytical and clinical diagnostics, particularly in immunochemical assays, such as chemiluminescent immunoassays (CLIA) and enzymatic chemiluminescent immunoassays (ECLIA) [1,2,3,4]. Compared to conventional UV-Vis absorbance-based methods, including enzyme-linked immunosorbent assays (ELISA), chemiluminescent detection offers substantially higher sensitivity, lower reagent consumption, and simplified assay protocols, while maintaining relatively low operational costs [5]. Detection limits of biological analytes such as antigens, antibodies, hormones, and other biological molecules can be easily achieved in the sub-femtomolar concentration range (<10^−15^ M) using chemiluminescent systems, thus exceeding the sensitivity of most alternative analytical techniques [6,7]. Moreover, chemiluminescent methods eliminate the need for radioactive tracers, thereby avoiding issues associated with radioactive decay, waste disposal, and specialised laboratory infrastructure, while preserving comparable analytical performance.

Among various chemiluminogenic systems, organic luminol derivatives and acridinium-based compounds represent the most widely used emitters in immunodiagnostic applications today. Acridinium esters and related derivatives of N-substituted acridine-9-carboxylic acid have attracted particular attention due to their generally high CL quantum yields, rapid flash-type and easy-tunable emission kinetics, and compatibility with aqueous and biological environments [8,9,10,11]. In contrast to luminol-based systems, acridinium chemiluminogens do not require enzymatic or catalytic activation, resulting in reduced background signal and improved signal-to-noise ratios [12,13,14]. The emission process involves rapid oxidative decomposition of the acridinium chemiluminogenic salt (active ester, sulfonamide, cyanide, and others), leading to the formation of electronically excited acridone derivatives that emit visible light in blue with high efficiency [15,16].

Acridinium chemiluminescent labels have been successfully applied in a wide range of biomedical and analytical applications, including the detection of proteins, hormones, nucleic acids, viral antigens, and low-molecular-weight analytes [13,17,18,19,20,21,22,23,24]. Detection limits at the attomole level have been reported in optimised analytical systems [2,25,26]. Depending on their structural design, acridinium-based systems may function either as covalently bound chemiluminescent labels, in which emission occurs following oxidative cleavage of a leaving-group fragment, or as hybridization probes and indicators, where the luminescent moiety remains associated with the analyte, as in hybridization protection assays (HPA) [23]. These approaches differ significantly in their structural requirements and synthetic constraints. Systems based on leaving-group cleavage require precise structural optimisation to ensure both efficient emission of light and appropriate reactivity toward conjugation, which represents a key challenge in the design of new reagents of potential utility in modern luminescence biodiagnostics.

Despite their favourable analytical properties, acridinium ester chemiluminogens remain susceptible to undesirable side reactions, including hydrolysis and formation of non-emissive pseudobase intermediates under alkaline conditions, which may reduce CL efficiency and compromise reagent stability [9,27,28], at the same time limiting some uses in terms of environmental requirements. Consequently, the development of structurally optimised chemiluminescent labels with improved emission efficiency, stability, and conjugation properties remains an important objective in luminescent bioanalytics. Structural modification of the acridinium scaffold represents, besides environmental tailoring, a promising strategy for improving analytical performance. In particular, the introduction of conjugated linkers adjacent to the ester moiety may facilitate efficient formation of electronically excited emitters during oxidative decomposition. Furthermore, spatial separation between the chemiluminogenic core and the reactive conjugation site enables independent optimisation of emission characteristics and bioconjugation capability. Such molecular design provides increased flexibility in tailoring chemiluminescent labels with enhanced signal intensity, reduced background emission, and improved stability under analytical conditions. Although commercially available acridinium labelling reagents, such as Acridinium C2 NHS ester [29], are widely used in immunodiagnostic applications, further improvements in emission efficiency, stability, and structural versatility remain desirable. On the other hand, optimising labelling protocols may also substantially enhance the usability and performance of resulting conjugates. Therefore, the development of novel acridinium chemiluminescent labels incorporating optimised linker build and their location shows an important direction for advancing chemiluminescent bioanalytical methods.

The objective of the present study was to systematically consolidate and critically evaluate physicochemical, synthetic, and analytical knowledge concerning acridinium chemiluminescent labels developed and investigated in our research group over the past decade. The work is based entirely on original material, which has not previously been presented in a unified and comprehensive form. Although numerous studies have addressed the application of acridinium-based chemiluminogens in biomedical luminescent assays, and other reports have focused on the structural and physicochemical properties of model acridinium salts, these aspects are typically considered separately. To the best of our knowledge, no study has provided an integrated framework combining synthetic methodology and characterisation, conjugation chemistry, emission performance, and theoretical analysis of acridinium chemiluminogens within a single, coherent investigation. Such an approach is, in our opinion, crucial for understanding the relationships between molecular structure, microenvironment, and analytical performance. Accordingly, the present work encompasses the synthesis and structural characterisation of a series of acridinium chemiluminescent labels (**AL1**–**AL5**), their conjugation to a model immunochemical protein (anti-human IgG), and evaluation of their CL performance under immunoassay-compatible conditions. Emphasis was placed on examining the influence of structural variation, labelling stoichiometry, and microenvironmental factors, including surfactant-mediated micellar systems, on emission intensity, stability, and analytical performance. In addition, theoretical approaches were performed to provide deeper, mechanistic insight into the reaction pathways and to assess the influence of dielectric environment and ionic interactions on the energetics of emissive processes. Such a combined experimental and theoretical approach, in our opinion, provides a comprehensive physicochemical and analytical characterisation of acridinium chemiluminescent labels relevant to their application in modern chemiluminescent immunoassays.

## 2. Results and Discussion

The results presented below collectively address the structural determinants of chemiluminescence efficiency, the suitability of the synthesised compounds for covalent conjugation with biomolecules, and their analytical performance under conditions representative of immunochemical detection systems. In this work, the term “structural determinants of chemiluminescence efficiency” refers to molecular features of the acridinium emitter—such as electronic effects of substituents, linker attachment position, and spacer structure—that influence the kinetics of the chemiluminescent reaction and the efficiency of excited-state formation. Particular emphasis was placed on elucidating how structural variation within the acridinium chemiluminogen and the local reaction microenvironment—including the presence of micellar surfactants—affects emission efficiency, signal stability, and detection sensitivity. To support and rationalise the experimental findings, theoretical calculations were performed to evaluate the electronic and energetic factors governing chemiluminescence reactivity and stability, thereby providing mechanistic insight into the observed structure-dependent behaviour.

**Figure 1:**
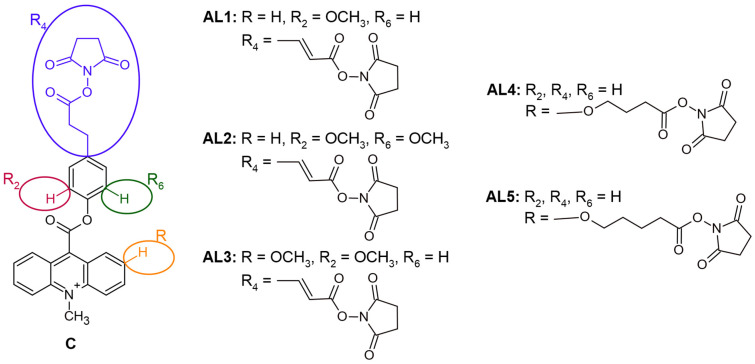
Structural formulas of the synthesised acridinium chemiluminogenic labels **AL1**–**AL5** and **C** [29] are investigated in this study. Their names and chemical characteristics are provided in the Supplementary Materials.

The investigated compounds comprise a series of originally developed acridinium chemiluminogenic labels (**AL1**–**AL5**), together with a commercially available reference reagent (**C**) [29] used as a benchmark in CL measurements. The chemical structures of **AL1**–**AL5** and **C** are presented in Figure 1, while their full chemical names and internal codes are provided in Table S1 in the Supplementary Materials. From a structural design perspective, these labels were intentionally developed to represent two different linker-attachment strategies employed in modern CL immunoassays: (i) functionalization within the leaving-group (phenoxy/aryl) moiety and (ii) functionalization introduced via the acridinium core. These complementary design concepts enable systematic evaluation of how the position and structural characteristics of the linker influence chemiluminescence efficiency, conjugation behaviour, and overall analytical performance.

The obtained compounds **AL1**–**AL3** and the reference compound **C** constitute a series of acridinium labels in which the reactive conjugation site is located within the leaving-group fragment. These derivatives differ in the nature of the unsaturated spacer (X = CH_2_–CH_2_ or CH=CH) and in the substitution pattern on the aromatic ring (R = H or OCH_3_), enabling systematic evaluation of structure–property relationships within this conjugation topology. Modulation of spacer rigidity and electronic effects within the leaving-group moiety is expected to influence both biomolecule coupling efficiency and the chemiluminescence-generating step, as the leaving-group structure plays a critical role in the formation and decomposition of the high-energy intermediate responsible for light emission. All compounds were isolated as quaternary acridinium triflate salts (CF_3_SO_3_^−^), ensuring a consistent ionic form and allowing direct comparison of their physicochemical and chemiluminogenic properties. In contrast, **AL4**–**AL5** represent an alternative structural design in which the linker is introduced at position 2 of the acridinium core. This approach enables controlled variation in spacer length (n) and spatial separation between the chemiluminogenic acridinium unit and the terminal reactive group, allowing assessment of how attachment position and molecular flexibility affect chemiluminescence efficiency and conjugation behaviour.

The synthetic routes to **AL1**–**AL3** and **AL4**–**AL5** are presented in Schemes S1 and S2 (Supplementary Materials). Briefly, **AL1**–**AL3** were prepared via a multistep sequence involving the synthesis of protected phenylcarboxylic acid derivatives, esterification with appropriately substituted 9-chlorocarbonylacridine, deprotection, and activation as NHS esters. Final products were obtained as N-methylated triflate salts and purified to >92% purity prior to use in protein conjugation and chemiluminescence studies. **AL4**–**AL5** were synthesised starting from a 2-methoxy acridine carboxylic acid phenyl ester derivative, followed by deprotection of the 2-hydroxyl group and O-alkylation with a protected haloacid. Subsequent deprotection and NHS activation afforded the reactive labels, which were isolated as N-methylated triflate salts of comparable purity (>92%) and employed for conjugation and chemiluminescence evaluation.

To assess optimal conditions for preparation of IgG–acridinium conjugates, a series of labelling procedures was evaluated using different molar excesses of acridinium labels relative to IgG. The investigation protocols included the commercial reference methodology and modified procedures corresponding to emitter-to-protein molar ratios ranging from 5-fold to over 40-fold molar excess (Procedure I-VI, Table S2, Supplementary Materials). The resulting conjugates were purified under identical conditions, and their performance and usability were further evaluated in various CLIA immunoassay formats [30].

**Figure 2:**
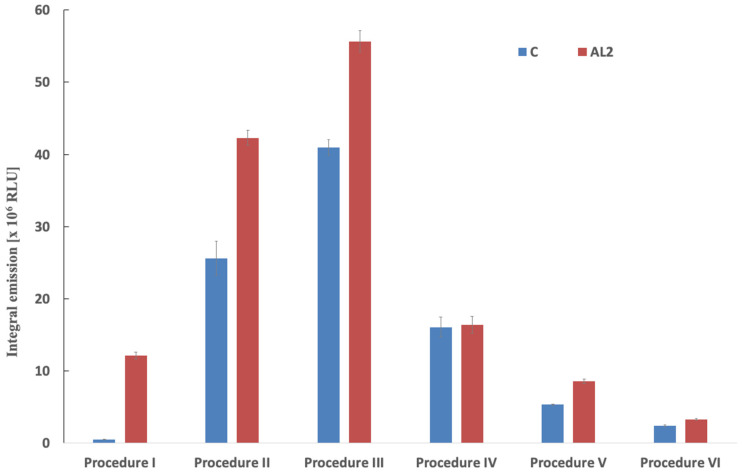
Effects of conjugation stoichiometry on integrated emission intensity of IgG–AL conjugates containing emitter **AL2** measured using the Fluoroskan Ascent FL luminometer. Procedure I corresponds to the commercial reference conjugation protocol [29]. Procedures II–VI represent modified labelling protocols using increasing molar excess of the acridinium label relative to IgG: 8.5× (Procedure II), 11.3× (Procedure III), 22.6× (Procedure IV), 34× (Procedure V), and 41.7× (Procedure VI). Detailed experimental conditions are provided in Table S2 (Supplementary Materials).

Before comparative evaluation, crude conjugates were subjected to size-exclusion purification to remove unreacted acridinium ester. Fraction selection was based on chemiluminescence screening of consecutive 1 mL fractions; a representative elution profile is presented in Figure S1 (Supplementary Materials). A well-defined emission maximum was observed, and only fractions corresponding to the principal conjugate peak were pooled for further analysis. As shown in Figure 2, the CL efficiency of the obtained conjugates exhibited a pronounced dependence on the applied labelling stoichiometry. Among the investigated variants, procedure III (Table S2, Supplementary Materials), corresponding to an approx. An 11-molar excess of acridinium label relative to IgG yielded the highest integrated emission intensity. This observation indicates that a moderate molar excess provides an optimal balance between labelling efficiency and preservation of favourable emissive properties of the conjugate, while maintaining the functionality of the investigated immunoglobulin. At lower labelling ratios, including the commercial reference procedure [29] and procedure II, reduced emission intensity was observed. This behaviour is likely consistent with incomplete occupation of available reactive sites on the protein surface, resulting in a lower number of chemiluminogenic centres per IgG molecule and consequently reduced signal generation. It is well established that the analytical performance of acridinium-based immunoconjugates (AL-IgG) is directly dependent on the degree of labelling, which determines the number of emissive centres per protein molecule [5,8,31]. In contrast, further increasing the molar excess of acridinium ester (procedures IV–VI) did not improve analytical performance and resulted in a progressive decrease in emission intensity. This finding indicates that excessive labelling adversely affects the chemiluminescence efficiency of the conjugates. Over-labelling can introduce steric constraints, alter protein conformation, thus promoting unfavourable interactions between neighbouring emitter moieties, facilitating non-radiative deactivation processes, and reducing the overall quantum yield of emission [5,8,32]. In addition, close spatial proximity of multiple acridinium emitters may promote intermolecular interactions that increase the probability of energy dissipation through non-emissive pathways. Similar effects have been reported for acridinium ester immunoconjugates, where optimal labelling ratios were required to achieve maximal chemiluminescence output while preserving both protein functionality and emission efficiency [5,8]. Importantly, the optimised procedure (procedure III) provided higher emission intensity than the commercial reference protocol, demonstrating that careful control of conjugation stoichiometry significantly improves the analytical performance of acridinium-based immunoreagents. These findings confirm that the degree of labelling represents a critical parameter governing the CL efficiency and must be optimised to achieve maximal signal output [5,8,32].

**Figure 3:**
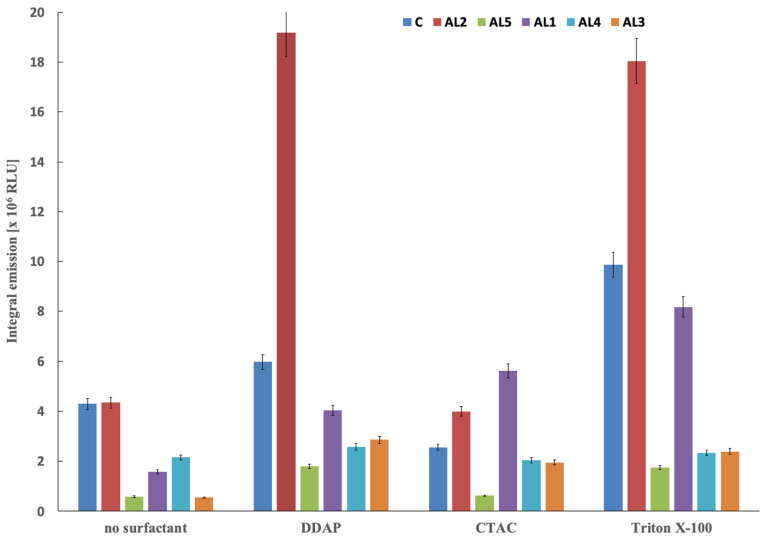
Influence of micellar surfactants (DDAPS, CTAC, Triton X-100) on integrated chemiluminescence intensity of IgG–acridinium conjugates containing representative emitters **AL1**, **AL2**, **AL4**, **AL5**, and commercial reference label **C** under immunoassay conditions (PBS, pH 6.5, r.t.). The emission was triggered in standard conditions (0.1% H_2_O_2_/NaOH system, pH = 11.5).

The influence of surfactant-mediated micellar environments on the chemiluminescence performance of IgG–acridinium conjugates was evaluated using surfactants of different characteristics: CTAC (cationic), DDAPS (zwitterionic), and Triton X-100 (nonionic) (Figure 3, Table S3, Supplementary Materials). Micellar surfactants employed in this work refer to surface-active agents that form micellar aggregates above their critical micelle concentration (CMC), thereby creating organised microenvironments that may influence the efficiency and kinetics of the chemiluminescence reaction. The surfactants used in this study were applied under conditions compatible with immunochemical assays; zwitterionic and nonionic surfactants are known to preserve antigen–antibody interactions, whereas adjustment of buffer pH was required in the case of CTAC to maintain stable immunocomplex formation. In the absence of surfactants, all conjugates exhibited measurable emission, confirming that the intrinsic chemiluminogenic reactivity of the acridinium labels is preserved following protein conjugation. Under these conditions, the commercial reference (**C**) and **AL2** provided the highest baseline signal, whereas **AL5** exhibited the lowest emission intensity, indicating substantial structure-dependent variation in CL efficiency in homogeneous aqueous solution. The introduction of micellar surfactants resulted in pronounced enhancement of chemiluminescence efficiency for most conjugates, although the magnitude of this effect strongly depended on emitter structure. The most pronounced amplification was observed for **AL2**, which exhibited an approximately three-fold increase in integrated emission in the presence of zwitterionic DDAPS and a similarly strong enhancement with Triton X-100, yielding the highest overall emission intensities among the investigated conjugates. **AL1** also demonstrated substantial susceptibility to micellar enhancement, particularly in the presence of Triton X-100 and CTAC, suggesting favourable partitioning of this emitter into organised micellar domains. **AL3** exhibited intermediate behaviour, showing moderate signal enhancement in the presence of DDAPS and Triton X-100. These observations suggest that structural features of **AL1** and A**L2** promote effective interaction with micellar environments, facilitating conditions favourable for efficient generation. In contrast, **AL4** exhibited only modest enhancement across all surfactant systems, while **AL5** showed consistently low emission intensity and limited response to micellar environments. This behaviour indicates that linker attachment via the acridinium core and/or increased spatial separation between the chemiluminogen and the protein may reduce the ability of the reactive centre to benefit from micellar organisation. Reduced micellar enhancement in these cases may result from less efficient localization of the acridinium moiety within the micellar interfacial region, where optimal polarity and local reactant concentration favour efficient formation of the emissive excited state. The commercial reference conjugate (**C**) exhibited moderate signal enhancement, confirming that micellar effects represent a general property of acridinium-based chemiluminescent systems rather than a feature specific to the newly developed derivatives. Among the investigated surfactants, DDAPS and Triton X-100 enabled the most effective signal enhancement, whereas CTAC produced a rather moderate effect. This behaviour is consistent with the ability of micellar systems to alter the local reaction microenvironment by increasing the effective concentration of reactive species, modifying local polarity, and stabilising high-energy intermediates involved in the chemiluminogenic transformations [2,31,33]. Partial localization of the acridinium moiety within the micellar interfacial region may reduce non-radiative deactivation pathways and facilitate more efficient formation and decay of the CL-generating intermediates. These results demonstrate that micellar organisation represents a critical environmental factor governing emission efficiency and that its effectiveness is strongly modulated by emitter structure.

The analytical performance of the investigated acridinium labels (**AL1**–**AL5** and reference compound **C**) was further evaluated using four luminometric platforms (Ascent FL, Centro XS3, Lumat3, and EnSpire) under both surfactant-free and micellar conditions (Tables S4–S7, Supplementary Materials). The obtained calibration parameters demonstrate that both the reaction microenvironment and the detection system significantly influence assay sensitivity and linear dynamic range. In the Ascent FL system, the addition of surfactants resulted in a pronounced increase in calibration slopes for all **AL** derivatives, with sensitivity enhancements ranging from approximately two- to four-fold. The strongest effects were observed for **AL1** and **AL4** in the presence of Triton X-100 and CTAC. This increase in slope was accompanied by a substantial decrease in LOD values, reaching the 10^−10^ M level in selected systems, indicating that micellar environments significantly improve the efficiency of chemiluminescence signal generation and detection. This enhancement can be attributed to improved local organisation of reactants and stabilisation of reactive intermediates within micellar domains, which increases the probability of productive formation of the electronically excited acridone emitter. The observed increase in chemiluminescence intensity and analytical sensitivity under micellar conditions is consistent with previous reports demonstrating that the microenvironment plays a decisive role in determining emission efficiency in acridinium ester systems [8]. Cationic surfactants such as CTAC have been shown to enhance light output and alter emission kinetics, while micellar systems in general are known to influence local polarity, reactant distribution, and excited-state stabilisation [5,31,33]. Instrument-dependent differences in analytical performance were particularly evident in the Centro XS3 and Lumat3 systems. In the Centro XS3 platform, elevated intercept values and increased background signal—especially in the presence of DDAPS—were associated with higher LOD values despite relatively high calibration slopes. This behaviour is consistent with the fundamental dependence of detection limits on blank signal variability and calibration sensitivity, where increased baseline noise directly limits achievable analytical sensitivity [34]. The Lumat^3^ system exhibited generally higher detection limits and somewhat narrower linear concentration ranges under the investigated conditions. This behaviour may reflect detector response characteristics and background signal properties, which are known to influence the effective analytical sensitivity of luminometric measurements [5]. In contrast, the highest overall analytical sensitivity was achieved using the EnSpire platform, where LOD values consistently reached the 10^−10^ M range. Under these conditions, differences between surfactant-free and micellar systems became less pronounced, indicating that when instrumental sensitivity approaches the limits achievable under the investigated conditions, instrumental characteristics may represent a dominant factor influencing overall analytical performance. These observations reveal the importance of both chemical and instrumental optimisation in achieving maximal sensitivity in chemiluminescent detection systems.

**Figure 4:**
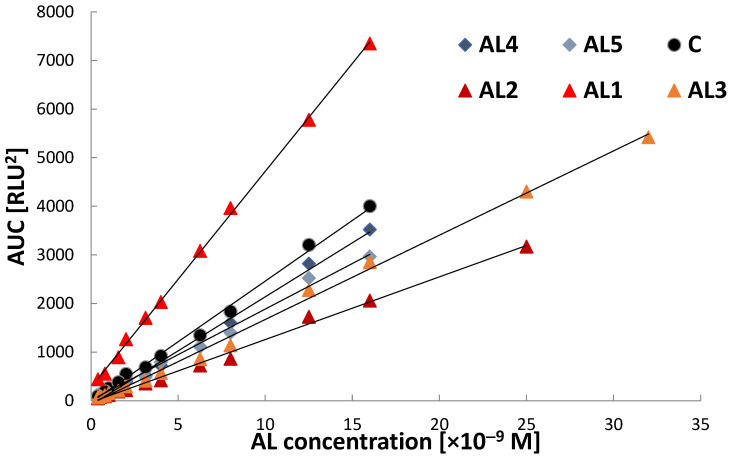
Calibration graphs for the investigated acridinium labels (**AL1**–**AL5**) and the commercial reference compound **C** measured using the Fluoroskan Ascent FL luminometer under surfactant-free conditions. Integrated chemiluminescence signal (AUC) is plotted as a function of acridinium label concentration.

**Figure 5:**
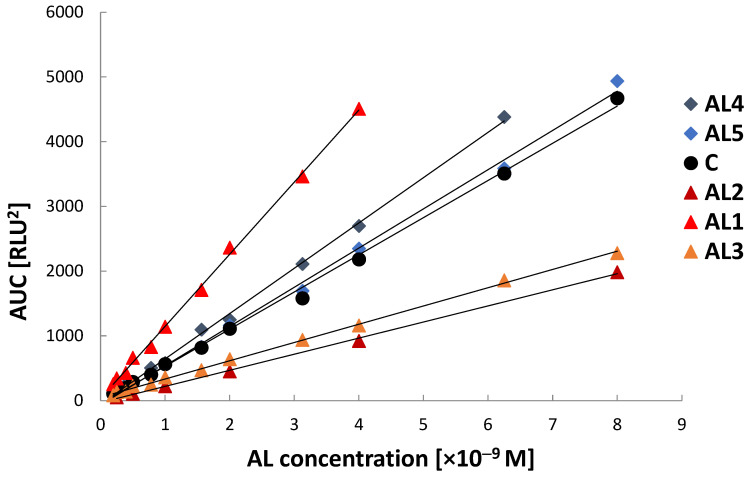
Exemplary calibration graphs recorded for investigated acridinium labels on the Ascent FL apparatus using Triton X-100 as surfactant.

The graphical calibration plots (Figure 4 and Figure 5) illustrate the concentration-dependent overall CL signal response of the investigated acridinium labels under surfactant-free conditions and in the presence of Triton X-100, respectively. In both cases, all emitters exhibit an approximately linear increase in integrated chemiluminescence signal (AUC) with increasing concentration within the investigated ranges, confirming a reliable quantitative response and proportional signal generation. Under surfactant-free conditions (Figure 4), clear differences in calibration slope are observed among the investigated derivatives, indicating structure-dependent variation in chemiluminescence efficiency. Among the studied emitters, **AL1** exhibits the steepest calibration slope, corresponding to the highest analytical sensitivity in an aqueous environment. **AL3** also shows a relatively strong signal response, whereas **AL4**, **AL5**, and the commercial reference compound (**C**) exhibit intermediate slopes. **AL2** displays the lowest calibration slope, indicating comparatively lower chemiluminescence efficiency under applied conditions.

In the presence of Triton X-100 (Figure 5), all emitters maintain linear calibration behaviour, confirming that micellar environments do not adversely affect the quantitative relationship between signal intensity and concentration. **AL1** continues to exhibit the highest calibration slope, indicating that its high chemiluminescence efficiency is preserved under micellar conditions. **AL4**, **AL5**, and the commercial reference compound (**C**) exhibit moderate slopes, while **AL2** and **AL3** express lower signal responses. Comparison of Figure 4 and Figure 5 demonstrates that the micellar environment influences the magnitude of the CL signal, while preserving linear calibration fit. These observations confirm that CL efficiency and analytical sensitivity depend on both emitter structure and reaction microenvironment, consistent with the known influence of local molecular organisation and intermediate stabilisation on acridinium emissive properties [8,31].

**Figure 6:**
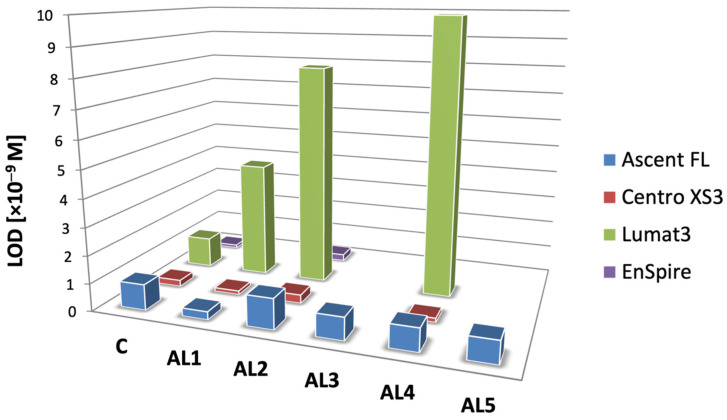
Comparison of detection limits (LODs) for **AL1**–**AL5** assessed for **ALs** with no surfactants present in the system (Tables S4–S7, Supplementary Materials).

To further illustrate the platform-dependent picture of analytical performance, the limits of detection obtained for all investigated labels are summarised in Figure 6. Although minor compound-dependent variations are observed, differences among luminometric platforms are more pronounced than differences between individual **AL** derivatives within a given instrument. In particular, the Lumat3 system consistently yielded higher detection limits, whereas the EnSpire and Centro XS3 platforms provided the lowest LOD values. This pattern indicates that instrumental factors—including detector sensitivity, signal acquisition characteristics, and background noise—represent critical determinants of achievable detection limits. These observations are fully consistent with the analytical definition of LOD as a function of calibration slope and blank signal variability, where increased baseline noise directly reduces detection sensitivity [34]. Taken together, these results demonstrate that while structural variation and micellar environments significantly influence chemiluminescence efficiency, the ultimate analytical sensitivity is determined by the combined effects of emitter structure, reaction microenvironment, and instrumental performance. This interplay between molecular design, chemical environment, and detector characteristics represents a key factor governing the practical analytical utility of acridinium-based chemiluminescent systems.

**Figure 7:**
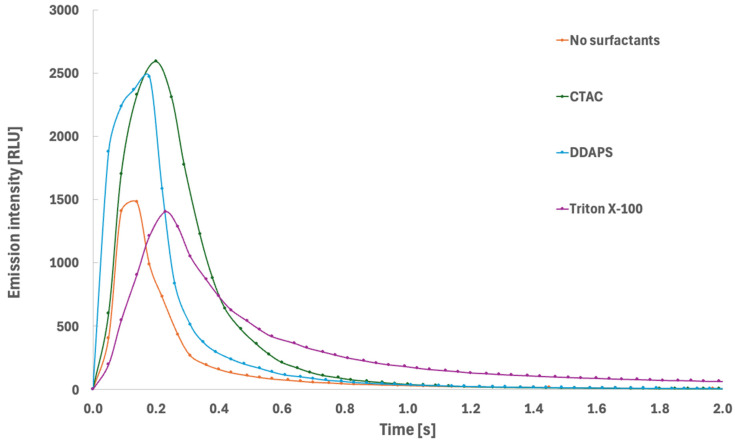
CL emission kinetics of exemplary acridinium label **AL1** in aqueous environment in the absence and in the presence of surfactants included in this work.

CL emission from acridinium labels is inherently time-dependent, and its analytical performance is governed not only by the total integrated emission, but also by the temporal characteristics of the emission profile. These kinetic characteristics reflect the rates of formation and decay of the electronically excited acridone derivative target emitter, as well as competing non-emissive pathways. To examine the influence of surfactant-mediated microenvironments on emission behaviour, the CL kinetic profiles of label **AL1** were recorded in aqueous buffer in the absence and presence of selected surfactants (Figure 7).

In the absence of surfactants, the emission exhibited a characteristic flash-type profile, with rapid signal rise followed by fast decay. This behaviour is typical of acridinium ester chemiluminescence in homogeneous aqueous solution, where rapid formation and decomposition of the high-energy intermediate result in a short-lived burst of emission. The relatively fast decay phase indicates efficient diffusion-controlled quenching and rapid depletion of reactive intermediates under these conditions. The introduction of surfactants resulted in substantial changes in both peak emission intensity and decay kinetics, indicating significant microenvironmental control over the CL reaction pathway. In particular, the presence of CTAC and DDAPS produced a pronounced increase in maximum emission intensity compared to the surfactant-free system. This enhancement likely indicates more efficient formation of the emissive excited state and reduced contribution of non-radiative deactivation pathways. Such behaviour is consistent with partial localization of the acridinium moiety and reactive intermediates within micellar interfacial regions, where increased local organisation and reduced exposure to bulk aqueous quenchers facilitate more efficient chemiluminescence generation. In contrast, the presence of Triton X-100 resulted in a distinctly prolonged emission decay phase. Although the peak emission intensity was comparable to or moderately higher than in aqueous solution, the signal persisted for significantly longer times, indicating an increased effective lifetime of emissive species. This behaviour suggests that micellar encapsulation alters the reaction kinetics by restricting diffusion and stabilising reactive intermediates, thereby slowing their consumption and extending the emission lifetime. Such kinetic stabilisation may also reduce the probability of initial non-radiative deactivation, contributing to improved overall emission efficiency.

Described observations demonstrate that micellar environments influence both the efficiency and kinetics of CL emission. Enhancement of peak intensity reflects improved efficiency of excited-state formation, whereas prolongation of emission lifetime is consistent with stabilisation of reactive intermediates and modification of reaction dynamics. From an analytical perspective, both effects contribute to improved signal detectability, either by increasing instantaneous signal intensity or by extending the temporal window available for signal acquisition. The observed kinetic modulation under micellar conditions is therefore fully consistent with the experimentally observed improvements in calibration sensitivity and detection limits and confirms that the reaction microenvironment represents a critical determinant of CL efficiency in acridinium-based detection systems.

To verify the quantitative reliability and analytical robustness of the developed chemiluminescence systems, all calibration models were subjected to statistical evaluation. Across all instrumental configurations, Pearson correlation coefficients exceeded 0.99, confirming excellent linearity over the investigated concentration ranges (Tables S4–S7, Supplementary Materials). These high correlation coefficients indicate a strong proportional relationship between analyte concentration and measured chemiluminescence signal, demonstrating a reliable and predictable analytical response of the investigated acridinium labels. Residual analysis further confirmed homoscedastic behaviour of the calibration data, indicating uniform variance of experimental errors across the concentration range. This observation supports the validity of the linear regression model and justifies the application of unweighted least-squares regression, in accordance with established recommendations for calibration model evaluation in analytical chemistry [35,36]. The absence of systematic deviation patterns in residual plots indicates that signal generation is governed by consistent reaction and detection processes, without evidence of concentration-dependent bias or nonlinear effects within the investigated analytical range.

To evaluate potential differences in analytical sensitivity between the developed labels and the commercial reference reagent (**C**), a one-way ANOVA approach was applied to compare calibration slopes across all instruments–label combinations (Table S8, Supplementary Materials). For most experimental configurations, no statistically significant differences were observed (*p* > 0.05), indicating that the developed acridinium labels provide analytical sensitivity better or comparable to that of the established commercial reagent **C**. Statistically significant differences were detected only for **AL2** in measurements performed on Centro XS3 and EnSpire luminometers (*p* = 0.0255 and 0.0129, respectively). The absence of consistent statistical differences across multiple platforms suggests that these isolated effects are primarily instrument-dependent rather than arising from systematic structural limitations of the investigated substrates. From an analytical perspective, these results confirm the reliability and quantitative applicability of the developed CL labels. The combination of excellent linearity, low detection limits, and statistically validated calibration models demonstrates that the investigated labels provide stable, predictable, and quantitatively reliable signal responses. Importantly, their analytical performance is comparable to that of a commercially established chemiluminescent reagent, confirming their full suitability for use in quantitative chemiluminometric immunochemical detection systems under the optimised experimental conditions.

To rationalise the experimentally observed differences in CL efficiency and analytical performance among the investigated acridinium labels, theoretical calculations at the TD-DFT level of theory were performed for selected derivatives differing in the position of linker attachment (Scheme S3, Supplementary Materials). Three various attachment modes were considered: substitution via the phenolic leaving group (**AL1**, **AL2,** and **AL3**), substitution at position 2 of the acridinium nucleus (**AL4** and **AL5**), and substitution at the endocyclic nitrogen atom (**AL6**). These structural motifs were selected based on established linker strategies employed in acridinium-based chemiluminogens and immunodiagnostic systems, where linker position is known to influence both chemical reactivity and emission efficiency and range [37,38,39]. The other important factors are associated with synthetic issues—step numbers, yields, and overall costs. The compound **AL6** was included in the computational studies as suggested by Natrajan et al. as the most efficient CL label representing the family of acridinium esters [38,39]. The results were also compared with the results of studies on the only commercially available reagent in the free form, referred to in this work as reference (**C**) [29].

**Figure 8:**
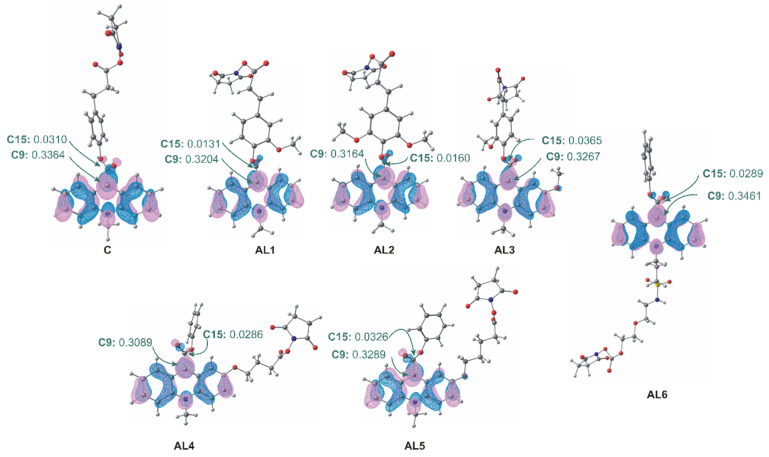
Computationally predicted Lowest Unoccupied Molecular Orbitals (LUMO) of investigated acridinium labels with the values of the LCAO coefficient of the p_z_ atomic orbital in their LUMO orbitals at the endocyclic C9 and the C15 carbonyl atom.

According to Frontier Molecular Orbital Theory, the spatial distribution of the lowest unoccupied molecular orbital (LUMO) determines regions most susceptible to nucleophilic attack, which represents the initial step of light-generating reaction [40]. Analysis of the calculated LUMO contours and LCAO coefficients revealed that the highest electron deficiency is localised at the endocyclic carbon atom C9 rather than at the carbonyl carbon (C15) (Figure 8). This result confirms that nucleophilic attack by peroxide or hydroxide ions is energetically favoured at the C9 position, in agreement with the established mechanistic model of acridinium ester chemiluminescence [9,41,42]. The localization of the LUMO at C9 provides a direct electronic explanation for the observed chemiluminescent reactivity and confirms that structural modification of the linker, while affecting steric and environmental interactions, does not disrupt the fundamental electronic requirements for light generation. The computed thermodynamic parameters further confirm that formation of the electronically excited 10-substituted acridan-9-one emitter is energetically favourable in both gas-phase and aqueous environments (Scheme S4, Table S9, Supplementary Materials). The latter supports the intrinsic thermodynamic feasibility of the chemiluminescent pathway under experimentally relevant conditions. Representative optimised geometries of key intermediates and transition states (Figure S2, Supplementary Materials) illustrate the structural evolution of the reacting system and confirm the accessibility of the chemiluminescence-generating reaction coordinate.

**Figure 9:**
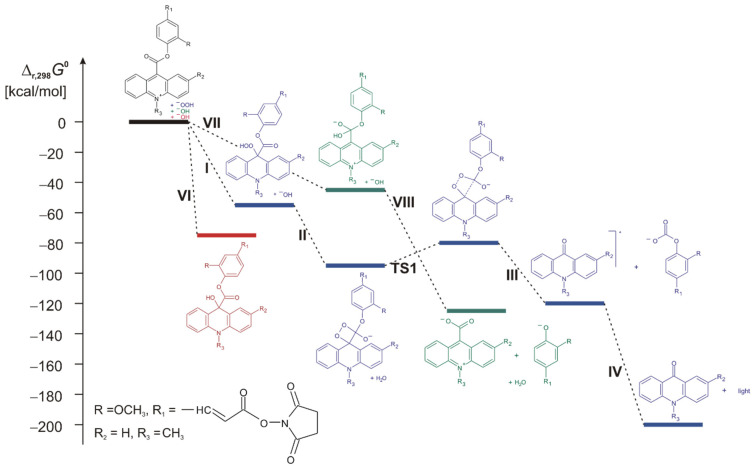
Gibbs free energy profiles of compound **AL1** (Scheme S3) for the process that account for chemiluminescence (blue structures), the formation of the so-called “pseudobase” (red structure), and the formation of the hydrolysis product—10-methylacridinium-9-carboxylate anions (green structures), calculated at the M06-2X level of theory.

Kinetic analysis revealed differences in activation barriers for the key CL-generating step (TS1), depending on the position of linker attachment (Figure 9). Higher activation energies were obtained for structures **AL2**, **AL3**, **AL4**, and the commercial (reference) compound **C** (18.3, 19.9, 19.4, and 17.6 kcal mol^−1^, respectively), whereas generally lower activation barriers were observed for structures **AL1**, **AL5**, and **AL6** (16.6, 15.2, and 13.1 kcal mol^−1^, respectively). These differences correspond to orders-of-magnitude variation in predicted reaction rates and indicate that linker attachment position can significantly influence the kinetics of excited-state formation and thus, emission. Lower activation barriers facilitate more rapid and efficient formation of the emissive acridone species, thereby increasing the probability of radiative decay and enhancing CL intensity.

These theoretical findings provide a mechanistic explanation for the experimentally observed differences in CL emission efficiency and micellar responsiveness among the investigated derivatives. Labels with lower calculated activation barriers are expected to exhibit more efficient chemiluminescence generation and greater sensitivity to environmental effects that stabilise reactive intermediates, such as micellar microenvironments. Conversely, higher activation barriers may limit the efficiency of excited-state formation and reduce overall emission intensity. In contrast, competing non-emissive pathways, including pseudo-base formation and hydrolysis, exhibited comparable energetic profiles across all investigated derivatives. This observation indicates that linker attachment position primarily influences the efficiency of the emissive reaction pathway rather than selectively promoting non-emissive decomposition. Consequently, differences in CL efficiency among the investigated labels are primarily associated with variations in the kinetics and energetics of excited-state formation rather than increased susceptibility to competing deactivation mechanisms. These conclusions are consistent with previously reported theoretical and experimental studies on acridinium chemiluminescence mechanisms and confirm the critical role of molecular structure in governing CL efficiency [9,41,42].

To rationalise the experimentally observed influence of micellar environments on chemiluminescence efficiency, semiempirical PM7 calculations combined with COSMO continuum solvation were performed using simplified dielectric models intended to approximate reduced-polarity microenvironments (ε = 4 and 10) alongside aqueous conditions (ε = 78.1). Reaction profiles were generated by constrained geometry optimizations along the C–C bond cleavage coordinate, and the highest-energy structures obtained along this coordinate were used as approximate indicators of the activation barrier (Figure S3A, Supplementary Materials). A gradual decrease in the calculated barrier was observed with decreasing dielectric constant. Relative to aqueous conditions (ε = 78.1), the barrier was reduced by approximately 0.88 kcal mol^−1^ at ε = 10 and by 2.05 kcal mol^−1^ at ε = 4. Additional calculations were performed in the presence of a nearby tetramethylammonium cation as a simplified electrostatic probe. Inclusion of this cation further lowered the calculated barrier in all environments (Figure S3B, Supplementary Materials), consistent with stabilisation of charge-separated configurations along the reaction coordinate (Figure S4, Supplementary Materials). It should be emphasised that the PM7/COSMO approach provides only a qualitative description of solvent and electrostatic effects and does not explicitly model specific micellar structures. Nevertheless, the internally consistent trends support the experimental observation that reduced polarity and local positive electrostatic fields may facilitate the formation of the emissive species and enhance chemiluminescence efficiency.

## 3. Materials and Methods

### 3.1. Synthesis of Acridinium Chemiluminescent Labels (**AL1**–**AL5**)

Acridinium chemiluminogenic labels **AL1**–**AL5** were synthesised using established multistep procedures based on commercially available acridine derivatives. The synthetic routes afforded the corresponding acridinium salts, which were fully characterised; detailed procedures and schemes are provided in the Supplementary Materials (Schemes S1 and S2).

Generally, labels **AL1**–**AL3** were obtained via a synthetic pathway involving the protection of the carboxyl group (as a benzyl ester) in a commercial aliphatic acid phenyl derivative, followed by its coupling with acridine-9-chlorocarbonyl derivatives. The resulting diester intermediates were subjected to acid-mediated deprotection to yield the corresponding acids, which were subsequently converted into N-hydroxysuccinimide (NHS) active esters using dicyclohexylcarbodiimide (DCC)-mediated activation. Final conversion into chemiluminescent acridinium salts was achieved by N-methylation using methyl trifluoromethanesulfonate or fluorosulfonate in anhydrous solvents in the presence of a sterically hindered base such as 2,6-di-tert-butylpyridine. The resulting acridinium triflate salts **AL1**–**AL3** were isolated by precipitation with diethyl ether and purified by reprecipitation with Et_2_O from absolute ethanol. Chemical yields ranged from ca. 78% to 91%, with final purities exceeding 90% as confirmed by RP-HPLC analysis. Detailed synthetic procedures and spectroscopic data are provided in the Supplementary Materials.

Labels **AL4** and **AL5** were synthesised using an alternative strategy involving the introduction of the linker moiety at position 2 of the acridine ring system. Initially, methoxy-substituted acridine precursors were converted into corresponding 2-hydroxyacridine derivatives via acid-mediated demethylation. Subsequent alkylation using protected ω-haloalkyl carboxylates enabled the introduction of linker chains of defined length. The benzyl-protected intermediates were then converted into NHS-activated esters following acid-mediated deprotection using carbodiimide-mediated activation. Final N-methylation using methyl trifluoromethanesulfonate yielded the target acridinium triflate salts **AL4** and **AL5**. The final compounds were isolated by precipitation, purified chromatographically, and characterised by NMR, MALDI-TOF MS, UV–Vis spectroscopy, and RP-HPLC. Overall yields for final steps ranged from 93% to 96%.

All synthesised acridinium labels were checked in view of build, applying a combination of ^1^H NMR spectroscopy, MALDI-TOF mass spectrometry, UV–Vis spectroscopy, and reversed-phase high-performance liquid chromatography (RP-HPLC), as appropriate for each compound. The obtained analytical data confirmed the assumed identity and high purity of the synthesised compounds. Detailed synthetic procedures, reaction conditions, and available spectroscopic and analytical data are provided in the Supplementary Materials.

### 3.2. Conjugation of Acridinium Labels to IgG and Evaluation of Chemiluminescence Performance

Chemiluminogenic acridinium labels (**AL1**–**AL5** and **C**) were dissolved in anhydrous dimethyl sulfoxide (DMSO) immediately prior to use to obtain 5 mM stock solutions. Commercial IgG (Goat Anti-Human IgG, ~150 kDa, Jackson ImmunoResearch Laboratories, Inc. (West Grove, PA, USA)) was dissolved in phosphate-buffered saline (PBS; 0.01 M sodium phosphate, 0.25 M NaCl, pH 7.5). The pH of the protein solution was adjusted to 8.0–9.0 using 0.01 M NaOH to provide mildly alkaline conditions favourable for NHS-ester-mediated coupling to lysine ε-amino groups. An appropriate aliquot of the acridinium label stock solution was added to the IgG solution to achieve a 10–12-fold molar excess of label relative to protein (optimal excess: 11.3-fold), as established in preliminary optimisation experiments (see Results and Discussion and Table S2, Supplementary Materials). The reaction mixture was stirred at room temperature (22–25 °C) for 30 min. Unreacted activated ester groups were quenched by the addition of 50 µL of 1% (*w*/*v*) lysine solution, followed by additional stirring for 15 min at room temperature. For labels requiring carbodiimide activation (compound type 3), conjugation was performed in the presence of equimolar amounts of 1-ethyl-3-(3-dimethylaminopropyl) carbodiimide hydrochloride (EDAC) and N-hydroxysuccinimide (NHS), using a 3–5-fold molar excess of the label relative to IgG.

Crude protein–label conjugates were purified by size-exclusion chromatography using Sephadex G-25 or G-50 as the stationary phase and acidic phosphate buffer (pH 3.5–5.0) as the mobile phase. Fractions (1 mL) were collected and screened for CL intensity using a plate luminometer Ascent FL). Fractions exhibiting the highest emission intensity were pooled and further purified by ultrafiltration using centrifugal filter units (30–100 kDa molecular weight cut-off). The conjugates were washed with slightly acidic distilled water (pH 5–6) and isolated either as aqueous solutions or as dry material after lyophilization. Protein concentration in purified conjugate fractions was determined spectrophotometrically by measuring absorbance at 280 nm (UV–Vis) using an appropriate extinction coefficient for IgG. In selected cases, protein concentration was additionally verified using the Bradford assay [43]. Conjugates were stored at −20 °C until each use.

White polystyrene 96-well plates suitable for immunoassays (MaxiSorp White Multiwell Plates, ThermoFisher Scientific, Rochester, NY, USA) coated with human IgG antigen were incubated with serial dilutions of IgG–acridinium conjugates for 60 min at 37 °C. After washing and drying, chemiluminescence was initiated by sequential addition of 10–50 µL of 0.1% (*w*/*v*) hydrogen peroxide (H_2_O_2_) in 0.01 M HNO_3_ containing 5 mM of one of the surfactants (CTAC, DDAPS, or Triton X-100, followed by 0.20–0.25 M NaOH. Total emission was recorded for 3–30 s, depending on the emission profile of the label, ensuring collection of at least 99% of the emitted signal. Calibration curves were constructed by plotting integrated emission intensity against analyte concentration.

### 3.3. Chemiluminescence Measurements and Data Processing

#### 3.3.1. Preparation of Stock and Working Solutions

Stock solutions of acridinium labels (**AL1**–**AL5** and reference compound **C**) were prepared at a concentration of 5 mM in N, N-dimethylformamide (DMF or DMSO) and stored at 4 °C in small vials protected from light. Working solutions were subsequently prepared in 10 mL volumetric flasks by appropriate dilution in 10^−3^ M HCl. All concentrations reported in the calibration graphs correspond to final concentrations in the reaction mixture. For injector solutions, concentrations refer to the prepared reagent solutions prior to mixing in the measurement chamber. Serial dilutions were prepared freshly before each measurement series.

#### 3.3.2. Measurements Using Ascent FL Plate Luminometer

Working solutions (2 × 10^−7^ M in 10^−3^ M HCl) were dispensed in 100 µL aliquots into 96-well microplates. Two initial dilutions were pipetted into subsequent wells A1–A6 and A7–A12, followed by serial dilutions across rows B–F using 10^−3^ M HCl. Chemiluminescence was initiated by automatic injection of 100 µL of 0.06% (*w*/*v*) H_2_O_2_ in 0.01 M HNO_3_ (Dispenser 2), optionally containing surfactants (24 mM CTAC, 24 mM DDAPS, or 60 mM Triton X-100), followed by 100 µL of 0.2 M NaOH (Dispenser 1). Signal acquisition was performed for 100 data points at 20 ms intervals (total integration time 2 s), with detector sensitivity set to 1000 mV. For selected compounds exhibiting prolonged emission profiles (e.g., **AL3**), extended signal acquisition (500 data points at 20 ms intervals, total 10 s) was additionally performed to ensure complete signal integration.

#### 3.3.3. Measurements Using Centro XS3 (Berthold) Luminometer

Working solutions (4 × 10^−8^ M in 10^−3^ M HCl) were prepared and dispensed analogously to the Ascent FL protocol in 96-well microplates, with serial dilutions performed in 10^−3^ M HCl. Chemiluminescence was triggered by sequential injection of 100 µL of 0.06% H_2_O_2_ in 0.01 M HNO_3_ (with optional surfactants: 24 mM CTAC, 24 mM DDAPS, or 60 mM Triton X-100), 100 µL of 0.2 M NaOH. Total signal integration time was 2 s.

#### 3.3.4. Measurements Using Lumat3 Tube Luminometer

For Lumat3 measurements, working solutions (2 × 10^−7^ M in 10^−3^ M HCl) were analysed in polypropylene tubes. Two initial dilutions were prepared, followed by serial dilutions derived from these starting solutions. Each dilution was measured in sextuplicate. For each measurement, 1 mL of the analyte solution was placed in the tube. Chemiluminescence was initiated by injection of 1 mL of 0.06% H_2_O_2_ in 0.01 M HNO_3_ (with optional surfactants at 24 mM CTAC, 24 mM DDAPS, or 60 mM Triton X-100), followed by 1 mL of 0.2 M NaOH.

#### 3.3.5. Measurements Using EnSpire Multimode Plate Reader

Chemiluminescence measurements were performed using an EnSpire multimode plate reader (PerkinElmer, Shelton, CT, USA). Working solutions were prepared in 10^−3^ M HCl and dispensed over 96-well microplates (100 µL per well), followed by serial dilutions prepared in the same medium. Chemiluminescence was initiated by sequential injection of 100 µL of 0.06% (*w*/*v*) H_2_O_2_ in 0.01 M HNO_3_ (optionally containing surfactants: 24 mM CTAC, 24 mM DDAPS, or 60 mM Triton X-100) and 100 µL of 0.2 M NaOH. Signal acquisition was performed in top-reading mode with a total integration time of 2 s per well. Detector sensitivity settings were maintained constant within each measurement series to ensure comparability of calibration data.

### 3.4. Data Processing and Statistical Analysis

Calibration curves were constructed by plotting integrated CL intensity (relative light units, RLU) against the final analyte concentration. The linearity range of the proposed assays was evaluated using serial dilutions of stock solutions of the respective acridinium labels (ALs). Linear regression models were obtained using the least-squares method. Pearson’s correlation coefficients (r) were calculated as the covariance between concentration (x) and signal intensity (y) divided by the product of their standard deviations, as described previously [44,45]. Limits of detection (LOD) and limits of quantification (LOQ) were calculated according to the procedure described in our previous study [44] and based on the calibration slope and the variability of the blank signal. Homoscedasticity of the regression models was evaluated by visual inspection of residual plots versus analyte concentration and by application of the F-Snedecor test, following the methodology described by Almeida et al. [46]. Comparison of calibration slopes was performed using one-way analysis of variance (ANOVA), according to the procedure described by Burns et al. [47,48,49]. Each calibration point represents the mean of replicate measurements. All statistical analyses were carried out using IBM SPSS Statistics (version 29; IBM Corp., Armonk, NY, USA).

### 3.5. Theoretical Study

Unconstrained geometry optimisations of isolated molecules in the ground (S_0_) or excited singlet (S_1_, only in the case of 10-methyl-9-acridinone derivatives) electronic states were carried out at the DFT or TDDFT levels of theory [50] respectively, using the M06-2X functional [51] and 6-31G(d,p) basis set [52] implemented in the Gaussian 16 programme package [53]. After completion of each optimization, the Hessian (second derivatives of the energy as a function of the nuclear coordinates) was calculated to assess whether stationary structures had been obtained. The harmonic vibrational frequencies were then derived from the numerical values of these second derivatives and used to obtain the enthalpy and Gibbs’ free energy contributions at 298.15 K and standard pressure with the aid of a built-in computational program of statistical thermodynamics routines [54]. The solvent effect was included in the DFT and TDDFT calculations at the level of the Polarised Continuum Model (PCM) (UAHF radii were used to obtain the molecular cavity) [55,56]. The enthalpies (Δ_r,298_*H*^0^) and Gibbs’ free energies (free energies in the case of DFT(PCM)) (Δ_r,298_*G*^0^) of the reactions (r), as well as the enthalpies (Δ_a,298_*H*^0^) and Gibbs’ free energies (free energies in the case of DFT(PCM)) (Δ_a,298_*G*^0^) of activation (a) indicated in Scheme S4 were calculated by following the basic rules of thermodynamics [57]. The rate constants (_298_*k*^0^) for the gaseous phase reactions were obtained by applying the equation:

(1) k0298=RTNhexp[−Δa,298G0/(RT)]

resulting from the transition state theory, and the reaction completion time (_298_*τ*_99_) from the formula:

(2) τ99298=ln100/k0298

where *R*, *T*, *N*, and *h* denote the gas constant, temperature (298.15 K), Avogadro’s number, and Planck’s constant, respectively [57].

Semiempirical calculations were performed using the PM7 method [58] implemented in the MOPAC2016, version: 17.279L software package [59]. Solvent effects were approximated using the COSMO continuum model [60]. The structures of the dioxetane intermediate and the acridan spiroxy phenoxy alkoxide derivative (Figure S5, Supplementary Materials) were geometry-optimised at dielectric constants (ε) of 4.0, 10.0, and 78.1 (water). Reaction profiles were generated by performing constrained geometry optimisations along the C–C bond cleavage coordinate, starting from the minimised bond length (~1.6 Å) to bond dissociation (~2.2 Å) using a grid step of 0.05 Å. Additional calculations were carried out in the presence of either a tetramethylammonium cation (N(CH_3_)_4_^+^) or a methanesulfonate anion (CH_3_SO_3_^−^). Ions were initially positioned in the vicinity of the reactive C–C bond prior to geometry optimization. For comparative purposes, heats of formation were referenced to the minimised initial structure (ΔH_f = 0 kcal mol^−1^).

## 4. Conclusions

Five acridinium chemiluminescent labels (**AL1**–**AL5**), representing two distinct linker attachment strategies, were successfully synthesised, purified, and structurally characterised using spectroscopic and chromatographic methods. The developed compounds were efficiently conjugated to a reference antibody (anti-human IgG), yielding stable immunochemical reagents suitable for chemiluminescence-based detection in CLIA tests. Systematic evaluation demonstrated that both structural features of the acridinium label itself, as well as conjugation stoichiometry, significantly influence the emission efficiency of resultant reagents. Optimisation of the emitter-to-protein molar ratio was essential for achieving maximal signal output, whereas excessive labelling resulted in reduced emission intensity, consistent with steric and microenvironmental effects associated with over-substitution of the protein.

Chemiluminescence performance was strongly dependent on the features of the reaction medium. The presence of surfactants, especially zwitterionic DDAPS and neutral Triton X-100, produced substantial enhancement of emission intensity, confirming the important role of the microenvironment in modulating CL signal. Theoretical calculations provided mechanistic support for these observations, indicating that both dielectric properties and the proximity of ionic species influence the energetic profile of the chemiluminescence reaction pathway. The investigated labels demonstrated analytical performance comparable to that of the commercial reference reagent and remained fully compatible with standard immunochemical conjugation protocols used in chemiluminescent immunodiagnostics. The obtained results establish a coherent relationship between molecular structure, conjugation parameters, and environmental effects, providing a rational basis for the selection and optimisation of acridinium CL labels for immunodiagnostic applications. Statistical analysis of the data revealed their high-performance capabilities, both in the free form and in the form of a conjugate with the model antibody, IgG.

## Data Availability

All relevant data are included within the article and its Supplementary Materials.

## Supplementary Materials

The following supporting information can be downloaded at: https://www.mdpi.com/article/10.3390/molecules31061041/s1.
