# Supplementary material for: Acridinium Chemiluminogenic Labels—Synthesis, Analytical Performance, and Mechanism of Light Generation—A Comparison in View of Biomedical Diagnostics
Source: Molecules. 2026 Mar 20;31(6):1041. doi: 10.3390/molecules31061041 (PMC13029182; doi:10.3390/molecules31061041)
Supplement: Supplementary file 1 [file molecules-31-01041-s001.zip › molecules-4202552-supplementary.pdf]

Supplementary Materials

Acridinium Chemiluminogenic Labels—Synthesis, Analytical Performance, and Mechanism of Light Generation—A Comparison in View of Biomedical Diagnostics

Karol Krzymiński [1,*], Beata Zadykowicz [1,*], Justyna Czechowska [2], Paweł Rudnicki-Velasquez [3], Illia Serdiuk [4], Adam Sieradzan [1] and Lucyna Holec-Gąsior [5]

[1] Faculty of Chemistry, University of Gdansk, 80-308 Gdansk, Poland; adam.sieradzan@ug.edu.pl
[2] Cyprotex Discovery Ltd., No. 24 Mereside, Alderley Park, Nether Alderley, Cheshire SK10 4TG, UK; Justyna.Czechowska-Kryszk@cyprotex.com
[3] Department of Falsified Medicines and Medical Devices, National Medicines Institute, Chełmska 30/34, 00-725 Warsaw, Poland; p.rudnicki@nil.gov.pl
[4] Faculty of Mathematics, Physics and Informatics, University of Gdansk, Wita Stwosza 57, 80-308 Gdansk, Poland; illia.serdiuk@ug.edu.pl
[5] Department of Molecular Biotechnology and Microbiology, Faculty of Chemistry, Gda´nsk University of Technology, Narutowicza 11/12 Str., 80-233 Gda´nsk, Poland; lucholec@pg.edu.pl
* Correspondence: karol.krzyminski@ug.edu.pl (K.K.); beata.zadykowicz@ug.edu.pl (B.Z.)

Table S1. Structural formulas, chemical names and internal codes of acridinium CL labels **AL1–AL5** and commercial label **C** [30] investigated in this study.

**Internal code**	**Structure**	**Chemical name**
**C**	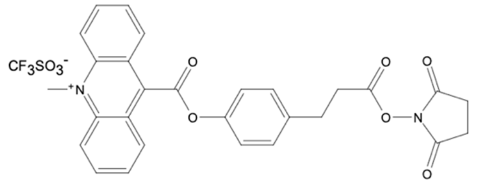	9-[(4-[3-[(2,5-dioxo-1-pyrrolidinyl)oxy]-3-oxopropyl]phenoxy)carbonyl]-10-methylacridinium 1,1,1-trifluoromethanesulfonate (MW = 632.56 g/mol CAS: 177332-37-5 [30])
**AL1**	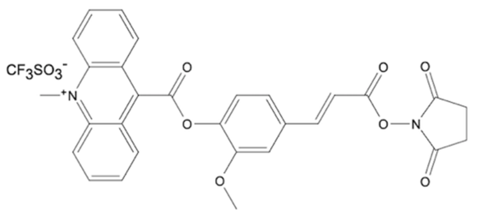	10-methyl-9-[(4-{(E)-3-[(2,5-dioxopyrrolidin-1-yl)oxy]-3-oxoprop-1-en-1-yl}-2-methoxyphenoxy)carbonyl]acridinium trifluoromethanesulfonate (MW = 660.57 g/mol)
**AL2**	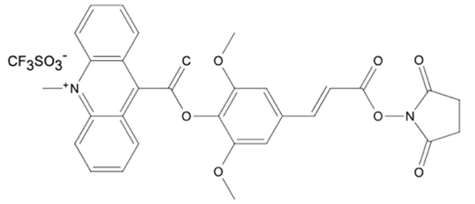	10-methyl-9-[(4-{(E)-3-[(2,5-dioxopyrrolidin-1-yl)oxy]-3-oxoprop-1-en-1-yl}-2,6-dimethoxyphenoxy)carbonyl]acridinium trifluoromethanesulfonate (MW = 690.60 g/mol)
**AL3**	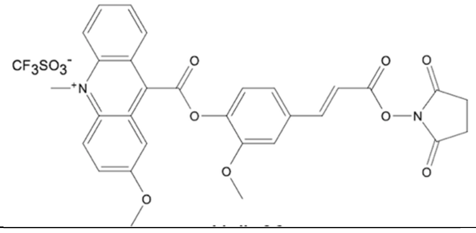	2-methoxy-10-methyl-9-[(4-{(E)-3-[(2,5-dioxopyrrolidin-1-yl)oxy]-3-oxoprop-1-en-1-yl}-2-methoxyphenoxy)carbonyl]acridinium trifluoromethanesulfonate (MW = 690.60 g/mol)
**AL4**	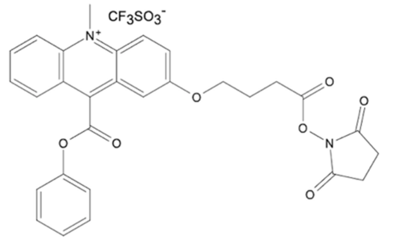	10-methyl-9-(phenoxycarbonyl)-2-[3-({[(2,5-dioxopyrrolidin-1-yl)oxy]carbonyl})propoxy]acridinium trifluoromethanesulfonate (MW = 662.59 g/mol)
**AL5**	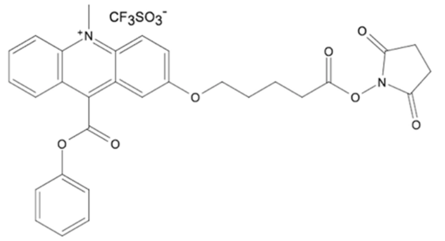	10-methyl-9-(phenoxycarbonyl)-2-[4-({[(2,5-dioxopyrrolidin-1-yl)oxy]carbonyl})butoxy]acridinium trifluoromethanesulfonate (MW = 676.62 g/mol)

Synthesis paths

Benzene ring-activated labels (AL1-AL3):

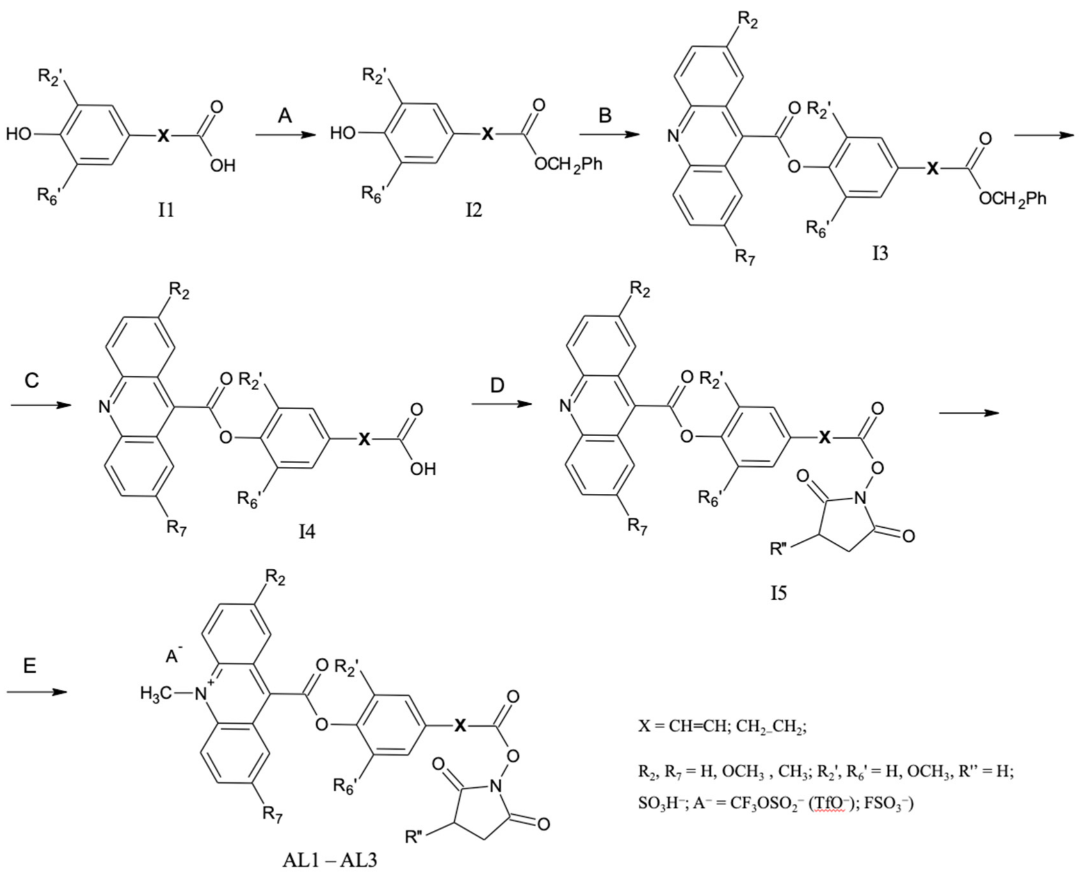

Scheme S1. General synthesis route to acridinium labels **AL1–AL3,** bearing spacer and the active (binding) group in the benzene ring. Reagents and reaction conditions are listed below the scheme.

Acridine ring-activated labels:

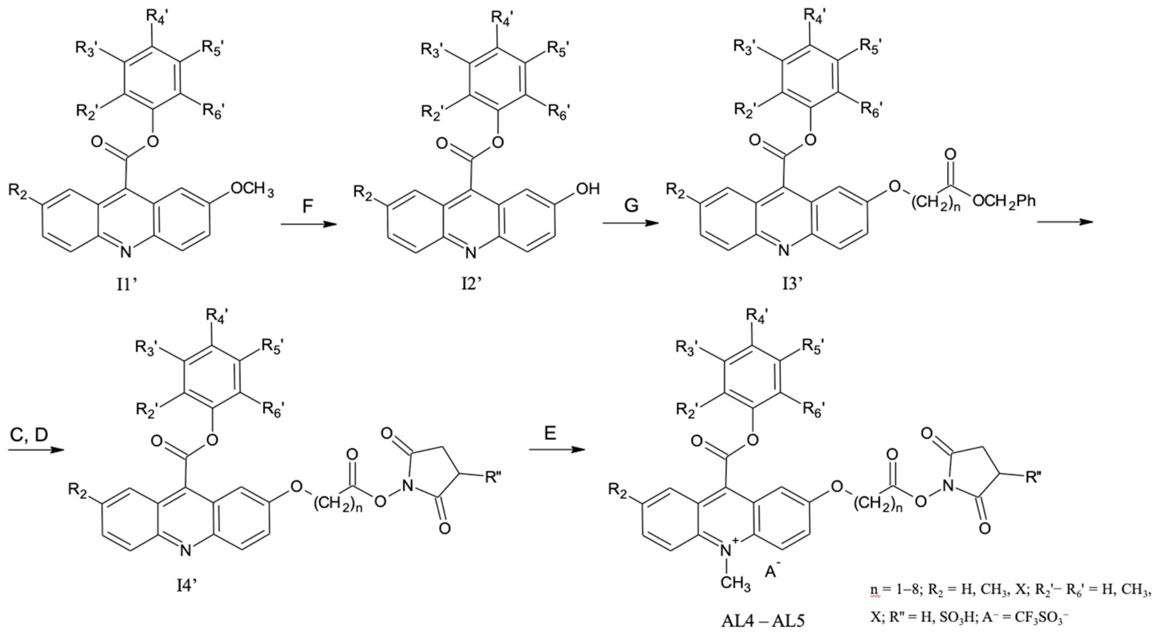

Scheme S2. General synthesis route to acridinium labels **AL4–AL5** bearing linker attachment at the position 2 of the acridinium ring.

Reagents and conditions (Scheme 1 and **2):**

A: $PhCH_2Cl$, KI, K_2CO_3, DMF, r.t., 5–30 g; **B**: 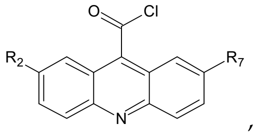 , Et_3N, DMAP, CH_2Cl_2, r.t.., 4–50 g. **C**: HBr/CH_3CO_2H, 20–50°C, 1–15 g. **D**: 1) DCC, DMF, 0–20°C, 0.5–2 g; 2) 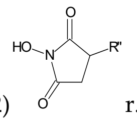 r.t., 6–20 g. **E**: $CF_3SO_3CH_3$ or FSO_3CH_3, 2,6-di-*tert*-butylpyridine, CH_2Cl_2 / $CHCl_3$, r.t. 1–15 g.

F: HBr in CH_3CO_2H, 80–150°C, 2–10 g.

G: 1) NaH, DMF, –20–0°C, 0.5–1 g; 2) $X(CH_2)_nCO2CH_2Ph$ (X = Cl, Br, 20–70°C, 1–10 g.

H: 1) $(COCl)_2$, CH_2Cl_2, 40–60°C, 0,5–3 g; 2) $AlCl_3$, CH_2Cl_2 or $CHCl_3$, temp. 0–25°C, 0,5–3 g; 3) OH^-, H_2O, 80–100°C, 5–30 g; 4) H_3O^+, H_2O, r.t.

I: 1) $SOCl_2$, 50–80 °C, 0.5–5 g; 2) 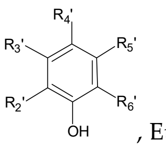 , Et_3N, DMAP, CH_2Cl_2, r.t., 4–50 g.

J: $NaBH_3R$ (R = H, CN), CH_2Cl_2, r.t., 0.5–5 g.

R_2, R_7 = H, CH_3, OCH_3,

R_2' – R_6' = H, CH_3, OCH_3

Procedures:

Benzene ring-substituted labels (AL1-AL3, Scheme 1)

Commercially available phenyl-substituted carboxylic acid derivative **I1** was converted by standard methods to the corresponding ester to protect the carboxyl functionality. In a typical treat, the carboxylic acid **I1** (1 mmol) was dissolved in dry dimethylformamide (DMF, 2 mL), followed by addition of anhydrous potassium carbonate (1 mmol), benzyl chloride (1.1 mmol), and potassium iodide (0.1 mmol). The reaction mixture was stirred at r.t. under anhydrous conditions for 5–30 h. After the completion, the contents were poured into excess cold water, and the products type **I2** were extracted with dichloromethane. The organic phase was washed with water, dried over anhydrous $MgSO_4$, filtered, and concentrated under reduced pressure to remove solvent.

The protected intermediates **I2** were next attached to acridine moiety to obtain aromatic esters type **I3** via esterification with derivatives of acridine. The benzyl esters type **I2** (1 mmol) were dissolved in dry dichloromethane (10 mL), and dry triethylamine (Et_3N) (3.3 mmol) and catalytic amount 4,4-dimethylaminopyridine (DMAP) were then added under Ar. 9-Chlorocarbonyl-acridine derivatives (1.1 mmol), prepared according to previously reported procedures [12, 40, 41], were then introduced to the mixture. The reaction was continued at r.t. for 4–50 h. After the completion, the solvent and excess of amine were removed under reduced pressure. The crude product was purified by recrystallization from methanol to afford intermediates type I3 in yields of 74%, 69-77%. Structural builds were checked using MALDI–TOF mass spectrometry and ^{1}H NMR spectroscopy. Removal of the benzyl protecting group was performed by acidic hydrolysis. Acridine ester intermediate I3 (1 mmol) was dissolved in 40% hydrobromic acid in acetic acid (5 mL) and stirred at 30–50 C for 1–15 h. The reaction mixture was then poured into excess ice-cold water, and the precipitated product was isolated by filtration and recrystallized from ethanol or ethanol/water mixtures to afford carboxylic acid intermediates **I4** in yields of 85%, 91%, and 90%, respectively. Representative ^{1}H NMR data for compound I4 (DMSO-d_6, 300 MHz, δ, ppm) were as follows: 4.07 (3H, s), 4.15 (3H, s), 6.68 (1H, d, J = 16.0 Hz), 7.44 (2H, m), 7.67 (1H, d, J = 16.0 Hz), 7.69 (1H, s), 7.89 (1H, t, J = 7.9 Hz), 7.95 (1H, t, J = 7.9 Hz), 8.13 (1H, d, J = 9.4 Hz), 8.25 (1H, d, J = 8.4 Hz), 8.36 (1H, d, J = 9.3 Hz), 8.59 (1H, d, J = 8.6 Hz), 12.5 (1H, br s).

Activation of the terminal carboxyl group was typically activated using carbodiimide (DCC)-mediated coupling with N-hydroxysuccinimide (NHS). Carboxylic acid intermediate **I4** (1 mmol) was dissolved in dry DMF and cooled in an ice bath. Dicyclohexylcarbodiimide (DCC, 1.2 mmol) was added, and the reaction mixture was stirred for 0.5–2 h, followed by addition of N-hydroxysuccinimide (1.2 mmol). The reaction was allowed to proceed at room temperature for 6–20 h. The mixture was diluted with dichloromethane, washed with water, dried over $MgSO_4$, filtered, and concentrated under reduced pressure. The product was purified by column chromatography on silica gel using 20–50% ethyl acetate in hexane or 1–5% isopropanol in chloroform as eluent to afford activated NHS ester intermediates **I5** in yields of 75%, 73%, and 78%, respectively. Representative ^{1}H NMR data for compound **I5** ($CDCl_3$, δ, ppm) were as follows: 2.81 (4H, s), 3.99 (3H, s), 6.55 (1H, d, J = 16.0 Hz), 7.22 (2H, m), 7.30 (1H, d, J = 8.6 Hz), 7.60 (2H, t, J = 7.9 Hz), 7.77 (2H, t, J = 7.2 Hz), 7.87 (1H, d, J = 16.0 Hz), 8.23 (2H, d, J = 8.8 Hz), 8.31 (2H, d, J = 8.8 Hz).

Final conversion of immediate precursors into chemiluminogenic acridinium labels was achieved by N-methylation of activated bases. Activated ester intermediate **I5** (1 mmol) was dissolved in dry dichloromethane, and polymer-supported 2,6-di-tert-butylpyridine (4 mmol) was added in

catalytic amounts under Ar. After approx. 10 min., methyl triflate (TfOMe) (5–10 mmol) was introduced, and the reaction mixture was stirred at r. t. for 1–15 h under anhydrous conditions. The reaction mixture was filtered through a 0.45 μm PTFE syringe filter and poured into excess dry diethyl ether. The precipitated acridinium triflate salts were isolated by filtration, washed with diethyl ether, and dried under reduced pressure to afford acridinium labels (final products) **AL1–AL3** in yields of 78- 91%. The products were obtained as analytically pure y (>92%) quaternary alts and stored under anhydrous conditions at lowered temperature (-20 deg C).

Representative spectroscopic data for **AL1** (CD_3CN, ^1H NMR, δ, ppm) were as follows: 2.84 (4H, s), 4.14 (3H, s), 4.87 (3H, s), 6.90 (1H, d, J = 16.0 Hz), 7.49 (1H, d, J = 8.2 Hz), 7.60 (1H, d, J = 8.2 Hz), 7.64 (1H, s), 8.03 (1H, d, J = 16.0 Hz), 8.16 (2H, t, J = 7.3 Hz), 8.51 (2H, t, J = 7.3 Hz), 8.70 (4H, m). The purity of the final compounds exceeded 90% as determined by RP-HPLC analysis (C18 column, acetonitrile/water with 0.1% TFA).

Acridine ring-activated labels **(AL4-AL5 (**Scheme S2)

2-Methoxy-substituted acridine derivatives type **I1′** [10, 28**]** were first converted into respective hydroxyacridine intermediates **I2′** via selective demethylation under strongly acidic conditions. In a typical procedure, compound I**1′** was placed in a pressure-resistant vessel and treated with an approx. 100-fold mass excess (w/w) of hydrobromic acid in acetic acid (HBr/CH_3CO_2H, 45/55 w/w). The reaction mixture was stirred at ca. 100°C for 3–5 h. After completion, the mixture was cooled and poured into ice-water, and the solution was neutralized using saturated sodium carbonate solution. The precipitated product was isolated by filtration, washed with water, and dried under reduced pressure. The resulting hydroxyacridine intermediates **I2′** were obtained in approx. yield of 65%. Representative spectroscopic data for compound **I2′** included m.p. (decomposition) at 273 °C and ^1H NMR (DMSO-d_6, δ, in ppm): 7.42 (1H, s), 7.43 (1H, s), 7.54–7.64 (5H, m), 7.74 (1H, t), 7.84 (1H, t), 8.20 (3H, br s).

Introduction of the spacer chains was achieved via O-alkylation of 2-hydroxyacridine intermediates (using protected ω-haloalkyl carboxylate derivatives. Hydroxyacridine intermediate **I2′** was dissolved in dry dimethylformamide and added dropwise under inert atmosphere (argon) to a cooled (−20 °C) mixture of slight excess of sodium hydride (NaH, oil susp.) in dry DMF. The mixture was stirred for approx. 30 min. at low temperature and then for an additional 30 min at r.t. A stoichiometric amount of protected ω-haloalkyl carboxylate derivative, such as benzyl esters of 4-bromobutyric or 5-bromovaleric acid, dissolved in dry DMF, was added gradually under inert atmosphere (Ar). The reaction mixture was then heated to 65–70°C and stirred for additional 3–5 h. After completion, the mixture was quenched with dilute hydrochloric acid solution (5–10%), and the product was extracted with toluene. The organic phase was washed with water, dried over anhydrous sodium sulfate, and concentrated under reduced pressure. The crude product was purified by column chromatography on silica gel using toluene/pyridine (10:1, v/v) as eluent to afford linker-functionalized intermediates **I3′** in yields of 86%- 89%, respectively. Representative ^1H NMR data for compound **I3′** ($CDCl_3$, δ ppm) were as follows: 2.21 (2H, quintet), 2.59 (2H, t), 3.49 (2H, t), 5.16 (2H, s), 7.39 (5H, s).

Removal of the benzyl protecting group and activation of the terminal carboxyl group were performed sequentially to obtain NHS ester intermediates **I4′**. Intermediates **I4′** were treated with HBr/AcOH reagent (33% solution) at 40–50°C for approx. 2 h. The reaction mixture was then poured into water, and the product was isolated by filtration, washed, and dried. Activation of the liberated carboxyl group was performed using N-hydroxysuccinimide under standard carbodiimide coupling

conditions, affording activated intermediates **I4′** in yields of 68%-70%. Representative ^{1}H NMR data for compound **I4′ (**13a ($CDCl_3$, δ ppm) were as follows: 2.38 (2H, quintet), 2.85 (4H, s), 2.95 (2H, t), 4.30 (2H, t), 7.38–8.30 (aromatic signals, m).

Final conversion of compound into chemiluminogenic salts was achieved by N-methylation of bases **I4′.** Activated ester intermediate **I4′** was dissolved in dry dichloromethane (DCM) and treated with 5-fold excess of freshly distilled methyl triflate in the presence of polymer-supported 2,6-di-tert-butylpyridine. The reaction mixture was stirred at room temperature under anhydrous conditions. The resulting acridinium triflate salts were isolated by precipitation with dry diethyl ether, re-precipitation by absolute ethanol-ethyl ether system, followed by filtration, washing with Et_2O, and drying under reduced pressure.

Final acridinium labels **AL4** and **AL5** were obtained in yields of 93% and 96%, respectively, as analytically pure triflate salts, suitable for and protein conjugation and chemiluminescent detection.

Representative analytical data for **AL4**:

^{1}H NMR (CD_3CN, δ ppm): 2.33 (2H, quintet), 2.76 (4H, s), 2.93 (2H, t), 4.42 (2H, t), 4.88 (3H, s), aromatic signals 7.49–8.63 ppm.

UV–Vis (CH_3CN): $\lambda max = 275$ nm ($\varepsilon = 6.79 \times 10^4$ dm^3 mol^{-1} cm^{-1}).

Representative analytical data for **AL5**:

^{1}H NMR (CD_3CN, δ ppm): 1.99 (4H, m), 2.76 (4H, s), 2.78 (2H, t), 2.93 (2H, t), 4.33 (2H, t), 4.84 (3H, s), aromatic signals 7.47–8.62 ppm.

UV–Vis (CH_3CN): $\lambda_{max} = 276$ nm ($\varepsilon = 6.82 \times 10^4$ dm^3 mol^{-1} cm^{-1}).

Analytical characterization of representative acridinium labels

AL1:

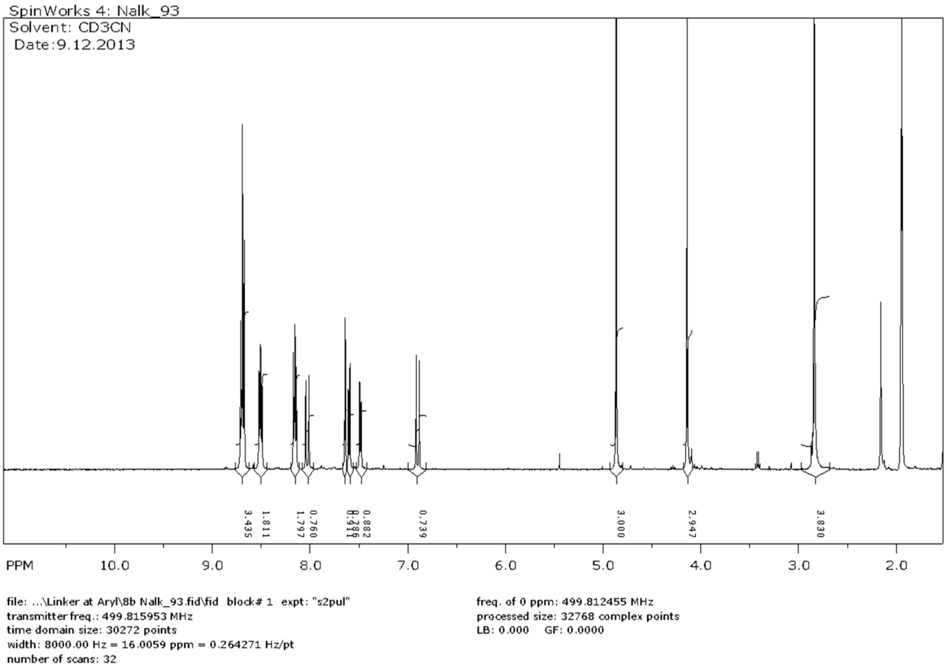

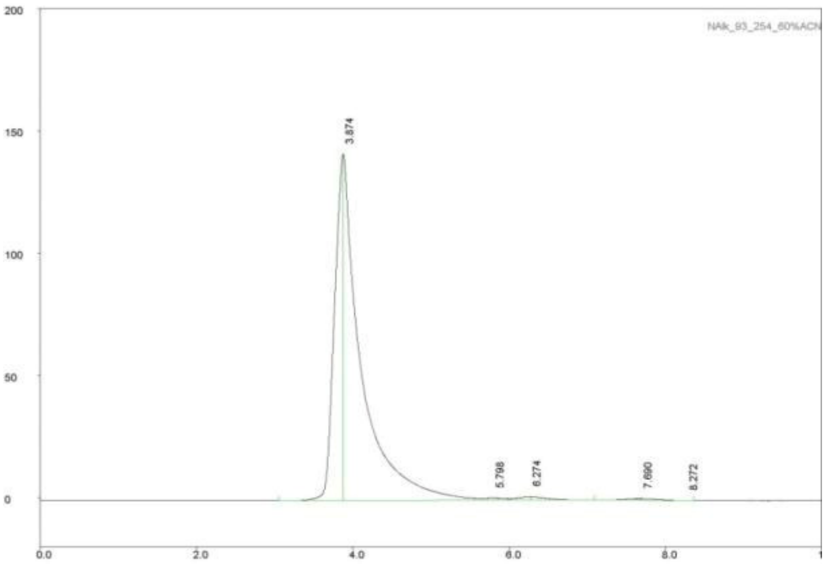

AL4:

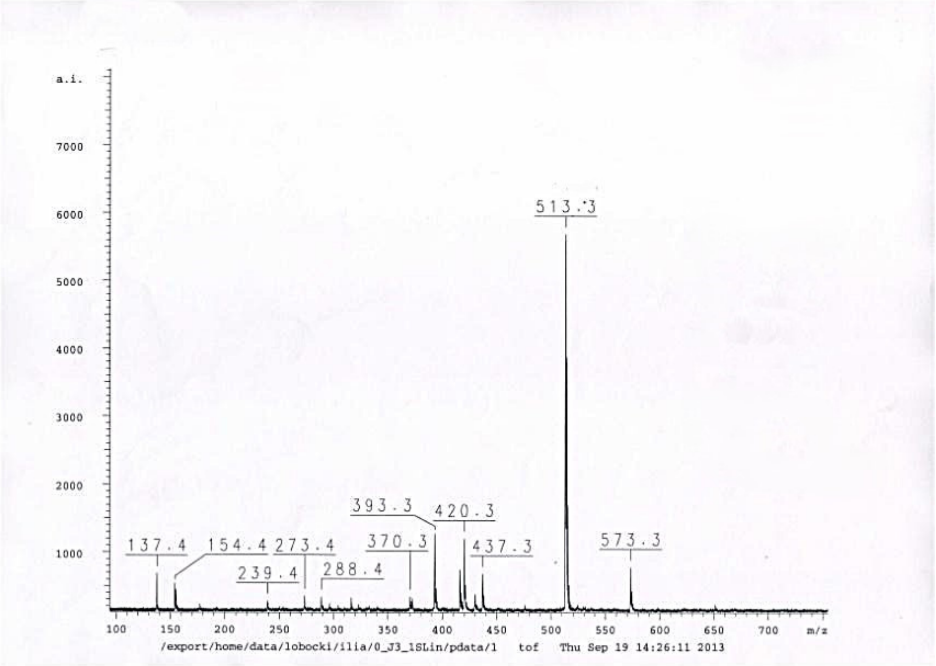
a.i.
7000
6000
5000
4000
3000
2000
1000
513.3
137.4
154.4
273.4
239.4
288.4
393.3
370.3
420.3
437.3
573.3
100
150
200
250
300
350
400
450
500
550
600
650
700
m/z
/export/home/data/lobocki/ilia/0_J3_1SLin/pdata/1 tof Thu Sep 19 14:26:11 2013

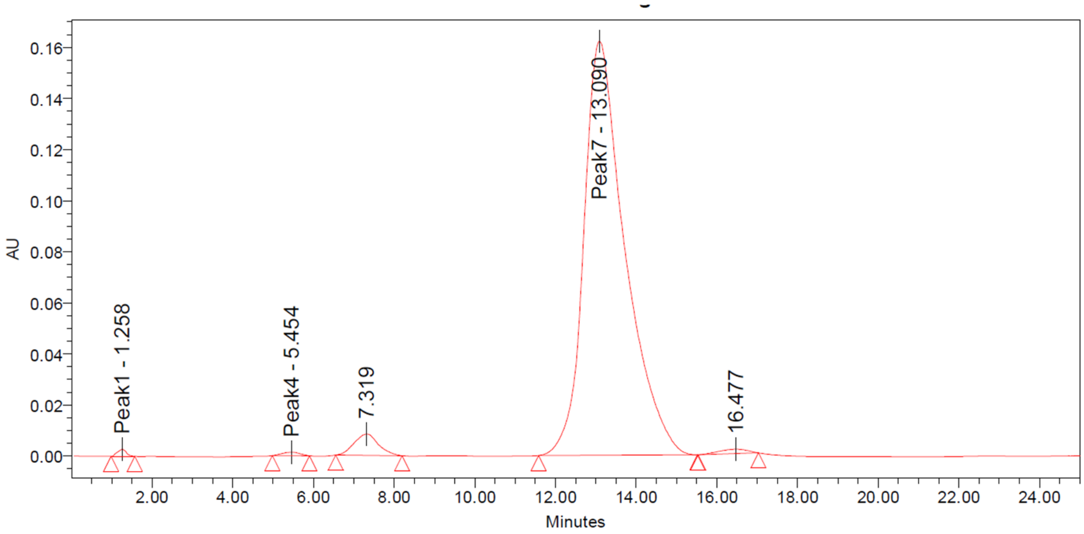
AU
0.16
0.14
0.12
0.10
0.08
0.06
0.04
0.02
0.00
Peak1 - 1.258
Peak4 - 5.454
7.319
Peak7 - 13.090
16.477
2.00
4.00
6.00
8.00
10.00
12.00
14.00
16.00
18.00
20.00
22.00
24.00
Minutes

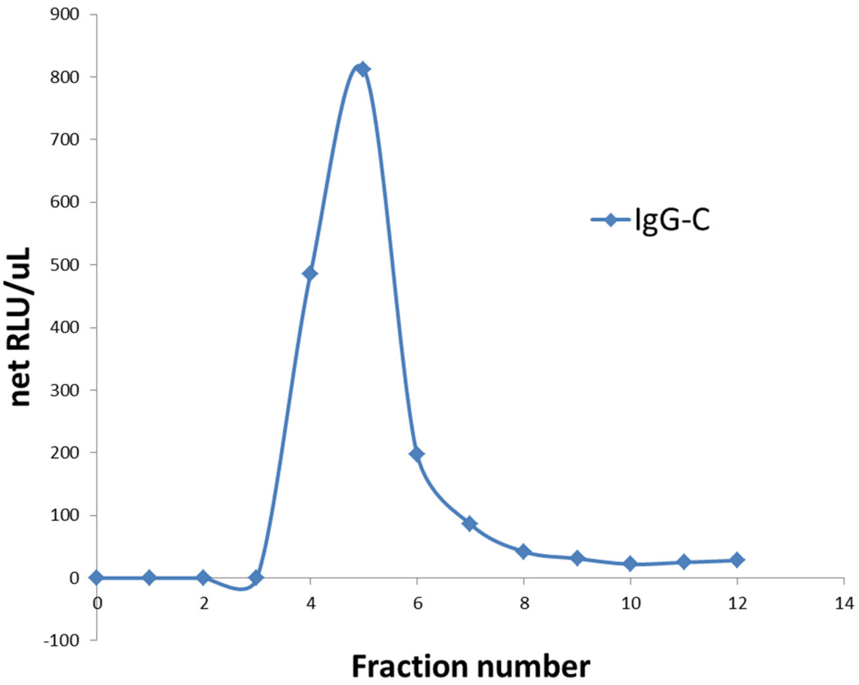

Figure S1. Exemplary labelling size-exclusion chromatogram for coupling reaction between **C** (the reference CL substrate) [30] and IgG antibody (AffiniPure GoatAnti-Human IgG H+L, minimal cross reactions, Jackson Immuno Research Labs, Inc., USA). Coupling was performed at r.t. under optimal conditions assessed for this process (Table S2) and conjugates were isolated using Sephadex G-25 for molecular filtration. The hottest fraction (#5 in Fig. S1) was used in CLIA tests reported in our other works [31].

Table S2. Optimization of IgG–AL conjugation conditions. Optimal conditions for labelling are marked in bold.

Procedure	IgG amount (mg)	IgG (mol)	AL (5 mM in DMF) [μL]	AL (mol)	AL:IgG molar ratio	pH	Lysine quench	Reaction time (AL)
I [30]	**21 μL IgG sol.**	—	**50**	**2.50×10^{-7}**	**~5× vs kit***	—	**200 μL (1%)**	**15 min**
II	0.24	1.6×10^{-9}	2.72	13.6×10^{-9}	8.5×	8.5	50 μL (1%)	30 min
III	0.18	1.2×10^{-9}	2.72	13.6×10^{-9}	11.3×	8.5	50 μL (1%)	30 min
IV	0.18	1.2×10^{-9}	5.44	27.2×10^{-9}	22.6×	8.5	50 μL (1%)	30 min
V	0.18	1.2×10^{-9}	8.16	40.8×10^{-9}	34×	8.5	50 μL (1%)	30 min
VI	0.18	1.2×10^{-9}	10	50×10^{-9}	41.7×	8.5	100 μL (1%)	30 min

**Relative to the manufacturer's recommended AE amount. All reactions were performed in acridinium labelling buffer; conjugates were purified by column chromatography and chemiluminescent fractions were collected.*

Table S3. Structural formulas, chemical names and internal codes of **surfactants** included in this study.

Internal code	Structural formula	Chemical name
**CTAC**	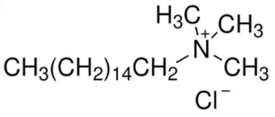	Hexadecyltrimethylammonium chloride; CAS: 112-02-7; MW = 320.0 g/mol
**DDAPS**	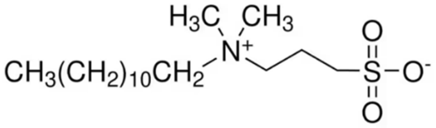	N-dodecyl-N, N-dimethyl-3-ammonio-1-propanesulfonate; CAS: 14933-08-5; MW = 335.55 g/mol
**TritonX-100**	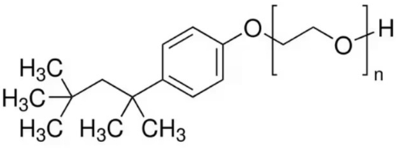 n = 9-10	2-[4-(2,4,4-trimethylpentan-2-yl) phenoxy] ethanol; CAS: 9002-93-1; MW = 602.80 g/mol (for n = 9), 646.85 g/mol (for n = 10)

Table S4. The parameters characterizing chemiluminescence assays for ALs obtained based on the calibration graphs measured with Ascent FL apparatus.

Ascent FL					
**Compound**	**Parameter**	**No surfactants**	**CTAC**	**DDAPS**	**Triton X-100**
**C**	Equation of the calibration graph (c – AL concentration [M])	2.49×10^{11} c – 32.4	3.60×10^{11} c + 107.60	3.85×10^{11} c + 43.92	5.74×10^{11} c – 43.85
	Linearity [$\times10^{-9}$M]	0.4 – 16.0	0.2 – 2.0	0.2 – 6.1	0.2 – 8.0
	Correlation coefficient (r)	0.9978	0.9994	0.9998	0.9988
	CV [%]	2.97	3.26	0.92	1.68
	LOD [M]	9.31×10^{-10}	1.11×10^{-10}	1.50×10^{-10}	2.54×10^{-10}
	LOQ [M]	30.96×10^{-10}	3.96×10^{-10}	4.78×10^{-10}	5.82×10^{-10}
**AL1**	Equation of the calibration graph (c – AL concentration [M])	2.86×10^{11} c + 20.21	6.05×10^{11} c + 57.47	5.99×10^{11} c + 53.95	1.11×10^{12} c + 39,68
	Linearity [$\times10^{-9}$M]	0.4 – 16.0	0.2 – 2.0	0.2 – 3.2	0.2 – 4.0
	Correlation coefficient (r)	0.9976	0.9989	0.9990	0.9984
	CV [%]	3.48	4.17	1.67	3.49
	LOD [M]	2.88×10^{-10}	1.51×10^{-10}	1.68×10^{-10}	2.13×10^{-10}
	LOQ [M]	9.68×10^{-10}	4.86×10^{-10}	5.66×10^{-10}	6.47×10^{-10}
**AL2**	Equation of the calibration graph (c – AL concentration [M])	1.0×10^{11} c – 29.947	3.33×10^{11} c + 99.13	2.89×10^{11} c + 68.05	2.49×10^{11} c – 28.45
	Linearity [$\times10^{-9}$M]	0.4 – 25.0	0.2 – 2.0	0.2 – 4.0	0.2 – 8.0
	Correlation coefficient (r)	0.9976	0.9973	0.9992	0.9994
	CV [%]	4.89	2.88	3.47	1.61
	LOD [M]	11.02×10^{-10}	0.98×10^{-10}	1.71×10^{-10}	1.25×10^{-10}
	LOQ [M]	37.89×10^{-10}	2.19×10^{-10}	5.71×10^{-10}	4.66×10^{-10}
**AL3**	Equation of the calibration graph (c – AL concentration [M])	2.0×10^{11} c – 60.931	7.65×10^{9} c + 26.16	2.21×10^{11} c – 13.16	2.82×10^{11} c + 52.76
	Linearity [$\times10^{-9}$M]	0.4 – 3.1	0.4 – 12.5	0.2 – 8.0	0.2 – 8.0
	Correlation coefficient (r)	0.9983	0.9994	0.9984	0.9993
	CV [%]	4.15	2.71	4.91	2.44
	LOD [M]	8.12×10^{-10}	3.08×10^{-10}	1.68×10^{-10}	2.08×10^{-10}
	LOQ [M]	25.07×10^{-10}	8.12×10^{-10}	5.47×10^{-10}	5.33×10^{-10}
**AL4**	Equation of the calibration graph (c – AL concentration [M])	2.22×10^{11} c – 80.23	8.36×10^{11} c + 110.80	5.07×10^{11} c + 106.61	6.99×10^{11} c – 54.63
	Linearity [$\times10^{-9}$M]	0.4 – 16.0	0.2 – 0.8	0.2 – 3.2	0.2 – 6.1
	Correlation coefficient (r)	0.9966	0.9979	0.9991	0.9992
	CV [%]	1.87	1.38	2.13	4.08
	LOD [M]	8.21×10^{-10}	2.61×10^{-10}	1.48×10^{-10}	2.37×10^{-10}
	LOQ [M]	26.96×10^{-10}	8.94×10^{-10}	4.81×10^{-10}	4.55×10^{-10}
**AL5**	Equation of the calibration graph (c – AL concentration [M])	1.87×10^{11} c + 12.19	6.14×10^{11} c + 123.0	4.19×10^{11} c + 161.92	6.06×10^{11} c – 65.91
	Linearity [$\times10^{-9}$M]	0.3 – 16.0	0.3 – 1.6	0.2 – 3.2	0.2 – 8.0
	Correlation coefficient (r)	0.9967	0.9970	0.9974	0.9987
	CV [%]	3.67	4.81	4.37	3.94
	LOD [M]	7.97×10^{-10}	0.88×10^{-10}	2.31×10^{-10}	2.43×10^{-10}
	LOQ [M]	25.43×10^{-10}	1.93×10^{-10}	5.66×10^{-10}	5.72×10^{-10}

Table S5. The parameters characterizing of the proposed chemiluminescence assay for ALs obtained based on the calibration graphs measured with Centro XS^3 apparatus.

Centro XS^3				
**Compound**	**Parameter**	**No surfactants**	**DDAPS**	**Triton X-100**
**C**	Equation of the calibration graph (c – AL concentration [M])	3.05×10^{14} c + 3.54×10^{5}	1.03×10^{14} c + 8.54×10^{6}	1.19×10^{15} c + 3.16×10^{5}
	Linearity [$\times10^{-10}$M]	1.0 – 64.0	1.8 – 65.3	1.2 – 64.0
	Correlation coefficient (r)	0.9914	0.9905	0.9956
	CV [%]	1.57	2.84	3.12
	LOD [M]	2.27×10^{-10}	4.38×10^{-9}	1.61×10^{-10}
	LOQ [M]	7.37×10^{-10}	14.57×10^{-9}	5.17×10^{-10}
**AL1**	Equation of the calibration graph (c – AL concentration [M])	1.71×10^{14} c + 3.47×10^{5}	2.02×10^{14} c + 8.49×10^{6}	1.68×10^{15} c + 1.04×10^{5}
	Linearity [$\times10^{-10}$M]	0.32 – 64.0	1.8 – 64.0	2.6 – 64.0
	Correlation coefficient (r)	0.9947	0.9866	0.9979
	CV [%]	1.82	3.89	4.08
	LOD [M]	1.32×10^{-10}	4.88×10^{-9}	0.91×10^{-10}
	LOQ [M]	5.12×10^{-10}	16.12×10^{-9}	3.33×10^{-10}
**AL2**	Equation of the calibration graph (c – AL concentration [M])	2.83×10^{13} c + 3.52×10^{5}	2.49×10^{14} c + 7.82×10^{6}	9.29×10^{14} c + 8.83×10^{4}
	Linearity [$\times10^{-10}$M]	1.0 – 64.0	1.8 – 64.0	1.2 – 64.0
	Correlation coefficient (r)	0.9922	0.9932	0.9981
	CV [%]	2.07	3.12	2.68
	LOD [M]	3.07×10^{-10}	5.81×10^{-9}	1.88×10^{-10}
	LOQ [M]	10.27×10^{-10}	19.27×10^{-9}	6.37×10^{-10}
**AL4**	Equation of the calibration graph (c – AL concentration [M])	4.15×10^{13} c + 3.88×10^{5}	4.56×10^{14} c + 8.22×10^{6}	1.42×10^{15} c + 2.61×10^{5}
	Linearity [$\times10^{-10}$M]	1.0 – 64.0	1.8 – 64.0	2.6 – 64.0
	Correlation coefficient (r)	0.9911	0.9915	0.9989
	CV [%]	2.64	2.88	1.89
	LOD [M]	1.48×10^{-10}	3.38×10^{-9}	1.91×10^{-10}
	LOQ [M]	4.78×10^{-10}	10.91×10^{-9}	6.22×10^{-10}

Table S6. The parameters characterizing of the proposed chemiluminescence assay for ALs obtained based on the calibration graphs measured with Lumat[3] apparatus.

Lumat[3]					
**Compound**	**Parameter**	**No surfactants**	**CTAC**	**DDAPS**	**Triton X-100**
**C**	Equation of the calibration graph (c – AL concentration [M])	1.04×10^{14} c – 6.95×10^{5}	3.36×10^{14} c + 1.07×10^{6}	4.75×10^{14} c + 5.78×10^{6}	2.60×10^{14} c – 1.01×10^{6}
	Linearity [$\times10^{-8}$M]	0.62 – 15.6	0.49 – 5.0	0.24 – 2.5	0.62 – 7.81
	Correlation coefficient (r)	0.9927	0.9970	0.9959	0.9941
	CV [%]	4.46	2.84	2.97	4.58
	LOD [M]	1.12×10^{-9}	2.53×10^{-9}	1.42×10^{-9}	5.02×10^{-9}
	LOQ [M]	3.72×10^{-9}	8.22×10^{-9}	4.79×10^{-9}	16.84×10^{-9}
**AL1**	Equation of the calibration graph (c – AL concentration [M])	1.51×10^{14} c – 7.20×10^{5}	4.68×10^{14} c + 2.76×10^{6}	5.02×10^{14} c + 1.10×10^{7}	4.86×10^{14} c – 7.14×10^{5}
	Linearity [$\times10^{-8}$M]	1.95 – 20.0	0.24 – 3.91	0.31 – 3.91	0.98 – 10.0
	Correlation coefficient (r)	0.9995	0.9985	0.9990	0.9994
	CV [%]	0.98	1.88	3.45	0.94
	LOD [M]	4.23×10^{-9}	1.26×10^{-9}	1.02×10^{-9}	2.21×10^{-9}
	LOQ [M]	14.07×10^{-9}	4.19×10^{-9}	3.43×10^{-9}	7.26×10^{-9}
**AL2**	Equation of the calibration graph (c – AL concentration [M])	8.07×10^{12} c + 2.17×10^{5}	2.82×10^{14} c + 1.08×10^{6}	1.47×10^{14} c + 4.68×10^{6}	1.79×10^{14} c – 5.23×10^{6}
	Linearity [$\times10^{-8}$M]	3.9 – 40.0	0.31 – 3.91	1.25 – 15.6	2.5 – 31.3
	Correlation coefficient (r)	0.9532	0.9917	0.9970	0.9753
	CV [%]	5.08	4.58	2.78	5.17
	LOD [M]	8.03×10^{-9}	3.07×10^{-9}	7.19×10^{-9}	28.88×10^{-9}
	LOQ [M]	26.79×10^{-9}	10.09×10^{-9}	23.98×10^{-9}	96.69×10^{-9}
**AL4**	Equation of the calibration graph (c – AL concentration [M])	7.04×10^{13} c – 2.50×10^{5}	6.72×10^{15} c + 2.25×10^{6}	2.89×10^{14} c + 1.65×10^{7}	2.58×10^{15} c – 7.61×10^{5}
	Linearity [$\times10^{-8}$M]	3.9 – 40.0	0.31 – 3.91	0.24 – 2.5	1.25 – 15.6
	Correlation coefficient (r)	0.9992	0.9925	0.9945	0.9974
	CV [%]	1.75	3.76	4.64	3.15
	LOD [M]	9.93×10^{-9}	2.77×10^{-9}	1.72×10^{-9}	6.74×10^{-9}
	LOQ [M]	32.88×10^{-9}	9.19×10^{-9}	5.58×10^{-9}	22.29×10^{-9}

Table S7. The parameters characterizing of the proposed chemiluminescence assay for ALs obtained based on the calibration graphs measured with EnSpire apparatus.

EnSpire				
**Compound**	**Parameter**	**No surfactants**	**DDAPS**	**Triton X-100**
**C**	Equation of the calibration graph (c – AL concentration [M])	6.77×10^{15} c – 3.84×10^{5}	9.09×10^{15} c + 3.31×10^{5}	8.08×10^{15} c + 5.07×10^{5}
	Linearity [$\times10^{-10}$M]	2.0 – 64.0	1.5 – 64.0	1.6 – 64.0
	Correlation coefficient (r)	0.9976	0.9977	0.9987
	CV [%]	2.42	3.05	2.28
	LOD [M]	1.12×10^{-10}	1.42×10^{-10}	1.48×10^{-10}
	LOQ [M]	3.47×10^{-10}	4.71×10^{-10}	5.07×10^{-10}
**AL2**	Equation of the calibration graph (c – AL concentration [M])	6.61×10^{14} c – 2.18×10^{4}	1.12×10^{15} c + 6.02×10^{5}	9.61×10^{14} c – 4.92×10^{5}
	Linearity [$\times10^{-10}$M]	1.8 – 50.0	1.5 – 64.0	1.5 – 64.0
	Correlation coefficient (r)	0.9949	0.9956	0.9963
	CV [%]	2.82	3.61	4.54
	LOD [M]	2.42×10^{-10}	2.33×10^{-10}	1.90×10^{-10}
	LOQ [M]	8.13×10^{-10}	7.52×10^{-10}	6.28×10^{-10}

Table S8. ANOVA table associated with simple linear regression analysis of data sets presented in Tables S2 – S5 (for analysis without surfactants; commercially available test – C [30] was used as reference standard).

Apparatus	Compound	F	p
**Ascent FL**	**AL1**	1.3485	0.2416
	**AL2**	1.8013	0.1932
	**AL3**	0.7043	0.4104
	**AL4**	0.1287	0.7232
	**AL5**	0.2830	0.6001
**Centro XS³**	**AL1**	1.3774	0.2498
	**AL2**	5.5264	0.0255
	**AL4**	2.8097	0.1041
**Lumat³**	**AL1**	2.7623	0.1187
	**AL2**	3.3351	0.0892
	**AL4**	2.7698	0.1183
**EnSpire**	**AL2**	7.1367	0.0129

F – F variance ratio; p – probability associated with F variance ratio

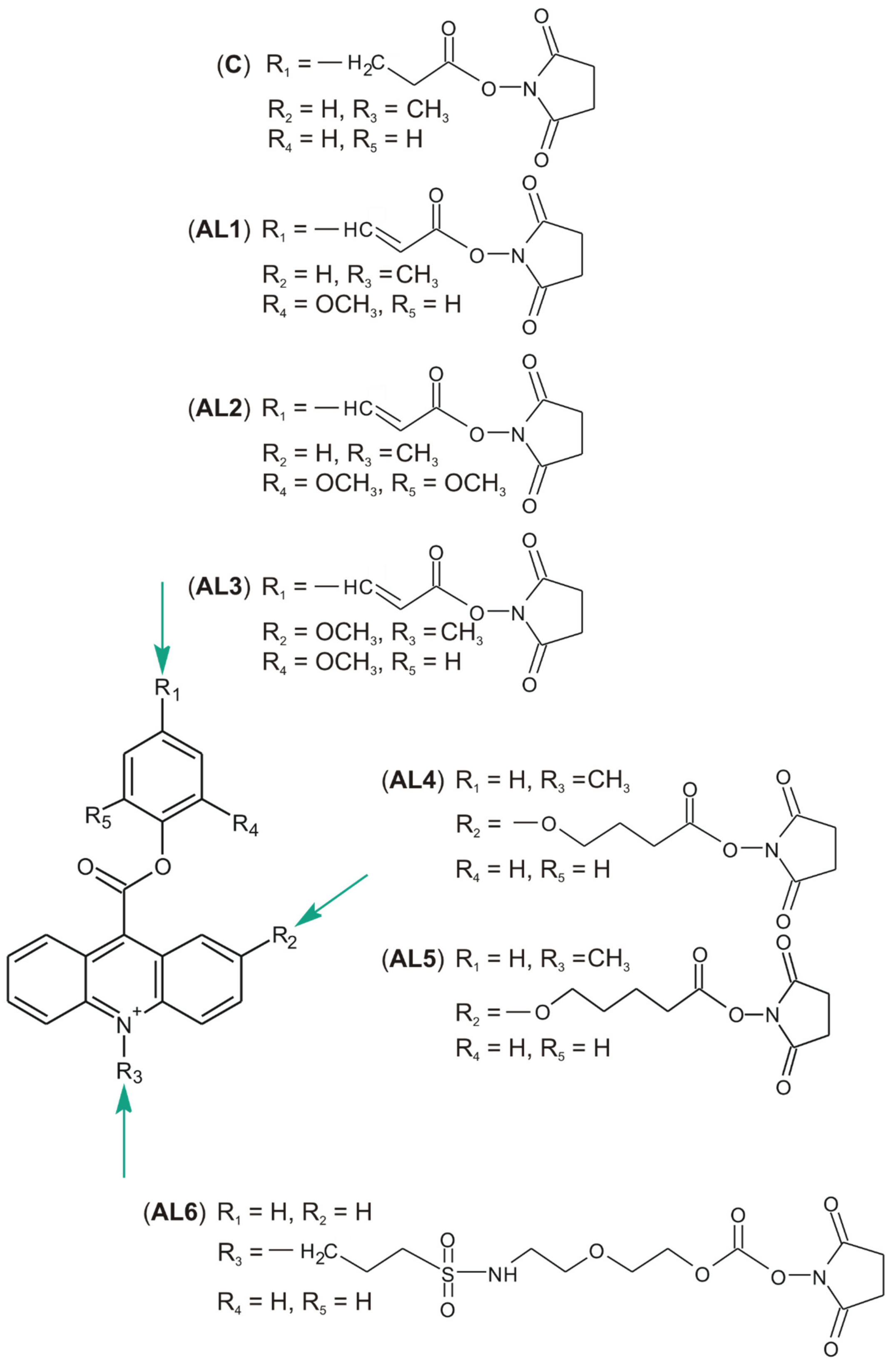

Scheme S3. Structural formula of the computationally investigated acridinium labels (ALs).

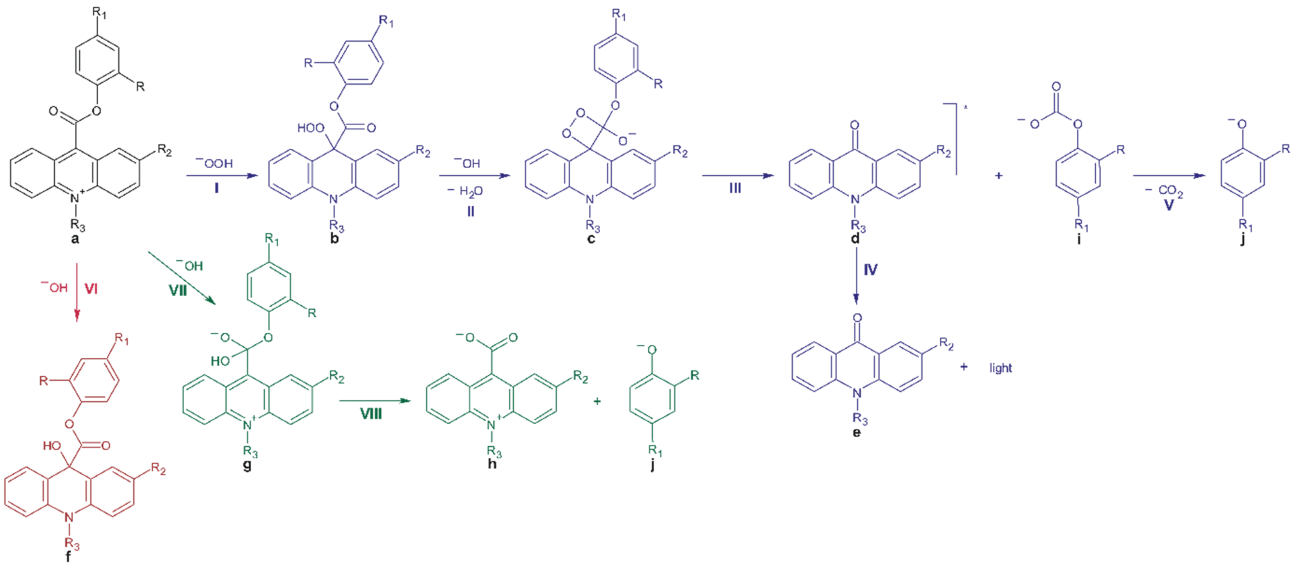

Scheme S4. Pathways of the reactions of investigated AL (Scheme S3) with OOH^- and OH^- (the process that account for chemiluminescence (blue structures), the formation of the so-called "pseudobase" (red structure) and the formation of the hydrolysis product – 10-methylacridinium-9-carboxylate anions (green structures), calculated at the M06-2X level.

Table S9. Thermodynamic and kinetic data of the elementary steps of the reactions of selected compounds with OOH^{-} and OH^{-} (Schemes S3 and S4) [a]

Thermodynamic data

step no. (Scheme S4)	compound no. (Scheme S3)	gaseous phase		aqueous phase	step no. (Scheme S4)	compound no. (Scheme S3)	gaseous phase		aqueous phase
		$\Delta_{r,298}H^{o}$	$\Delta_{r,298}G^{o}$	$\Delta_{r,298}G^{o}$			$\Delta_{r,298}H^{o}$	$\Delta_{r,298}G^{o}$	$\Delta_{r,298}G^{o}$
**I**	**AL1**	–180.3	–168.6	–57.4	**V**	**AL1**	3.8	–3.1	–0.4
	**AL2**	–174.5	–161.8	–55.4		**AL2**	1.4	–7.7	–0.3
	**AL3**	–173.4	–161.0	–53.5		**AL3**	8.0	–0.9	2.7
	**AL4**	–174.6	–162.0	–54.9		**AL4**	19.9	11.9	9.9
	**AL5**	–169.9	–159.9	–54.8		**AL5**	19.9	11.9	9.9
	**AL6**	–178.8	–166.8	–58.6		**AL6**	19.9	11.9	9.9
	**C**	–182.1	–169.1	–59.8		**C**	17.1	8.1	9.6
**II**	**AL1**	–81.7	–80.5	–39.4	**VI**	**AL1**	–206.1	–195.3	–76.4
	**AL2**	–83.1	–82.0	–38.8		**AL2**	–202.2	–191.1	–74.1
	**AL3**	–87.1	–86.6	–44.1		**AL3**	–201.1	–190.4	–74.2
	**AL4**	–78.6	–78.3	–41.4		**AL4**	–203.1	–192.2	–76.1
	**AL5**	–79.7	–79.3	–40.4		**AL5**	–198.3	–190.2	–75.5
	**AL6**	–70.8	–69.1	–31.0		**AL6**	–198.7	–188.0	–74.0
	**C**	–81.5	–75.2	–35.2		**C**	–201.8	–192.5	–74.9
**III**	**AL1**	–6.5	–22.0	–26.0	**VII**	**AL1**	–166.9	–156.0	–45.5
	**AL2**	–3.3	–17.2	–27.1		**AL2**	–163.9	–151.9	–45.2
	**AL3**	–5.5	–19.3	–33.3		**AL3**	–160.7	–150.7	–45.6
	**AL4**	–3.7	–18.8	–30.7		**AL4**	–159.8	–148.3	–42.7
	**AL5**	–2.3	–16.7	–30.6		**AL5**	–160.6	–150.9	–44.2
	**AL6**	–9.2	–24.7	–33.2		**AL6**	–165.4	–154.9	–47.1
	**C**	2.1	–18.9	–27.8		**C**	–164.5	–153.1	–44.6
**IV**	**AL1**	–80.3	–80.0	–82.4	**VIII**	**AL1**	–94.7	–108.6	–77.2
	**AL2**	–80.3	–80.0	–82.4		**AL2**	–92.5	–107.3	–75.8
	**AL3**	–83.0	–82.6	–77.5		**AL3**	–98.7	–112.0	–77.1
	**AL4**	–79.6	–78.6	–76.5		**AL4**	–72.6	–87.3	–68.1
	**AL5**	–80.9	–80.6	–77.5		**AL5**	–70.2	–85.4	–67.2
	**AL6**	–80.5	–80.2	–83.6		**AL6**	–69.8	–83.4	–65.2
	**C**	–80.3	–80.0	–82.4		**C**	–76.7	–92.5	–68.1

Kinetic data

step no. (Scheme S4)	compound no. (Scheme S3)	gaseous phase			aqueous phase
		$\Delta_{a,298}H^{o}$	$\Delta_{a,298}G^{o}$	$_{298}k^{o}$ ($_{298}\tau^{o}_{99}$)	$\Delta_{a,298}G^{o}$
**TS1**	**AL1**	11.9	12.3	5.9×10^{3} (7.8×10^{-4})	16.6
	**AL2**	15.0	14.6	1.2×10^{2} (3.9×10^{-2})	18.3
	**AL3**	15.0	14.2	2.6×10^{2} (1.8×10^{-2})	19.9
	**AL4**	12.3	13.0	1.9×10^{3} (2.5×10^{-3})	19.4
	**AL5**	10.0	10.2	2.1×10^{5} (2.2×10^{-5})	15.2
	**AL6**	9.1	9.3	9.5×10^{5} (4.8×10^{-6})	13.1
	**C**	15.1	13.5	7.3×10^{2} (6.3×10^{-3})	17.6

[a] $\Delta_{r,298}H^{o}$ and $\Delta_{r,298}G^{o}$ (both in kcal mol ^{1}) respectively represent the enthalpy and Gibbs' free energy (gaseous phase) or free energy (aqueous phase) of the reaction corresponding to a given step number at temperature 298.15 K and standard pressure; $\Delta_{a,298}H^{o}$ and $\Delta_{a,298}G^{o}$ (both in kcal mol ^{1}) respectively represent the enthalpy and Gibbs' free energy (gaseous phase) or free energy (aqueous phase) of activation at temperature 298.15 K and standard pressure; $_{298}k^{o}$ (in s^{-1}) and $_{298}\tau^{o}_{99}$ (in s) (Eqs. (1) and (2)) respectively denote the rate constant and the time after which the reaction is 99% complete.

Entity no. (Scheme S4)	Compound (Scheme S3)			
	**C**	**AL1**	**AL2**	**AL3**
**a**	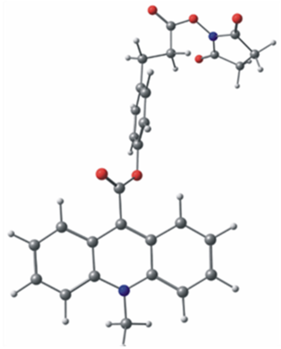	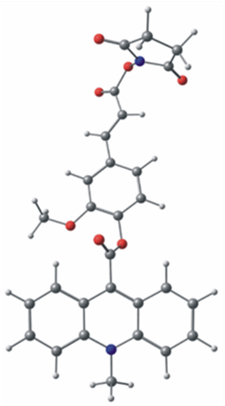	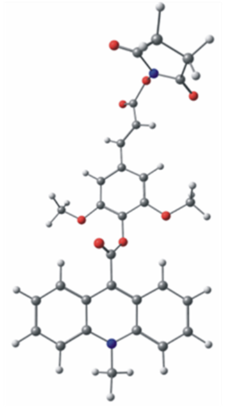	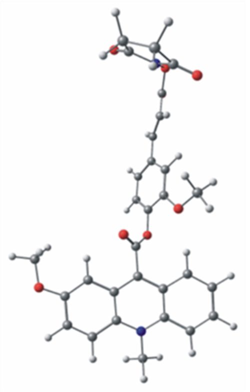
**b**	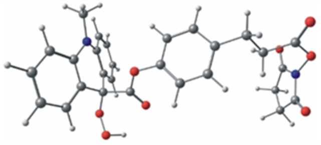	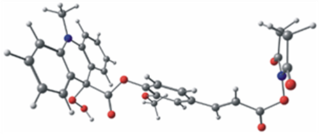	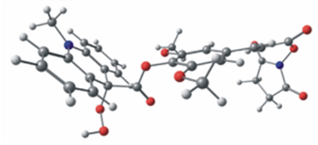	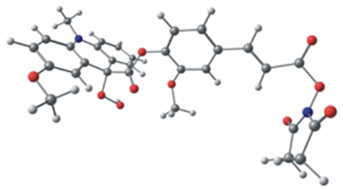
**c**	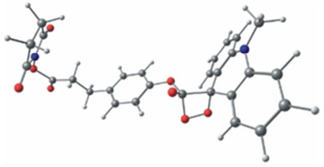	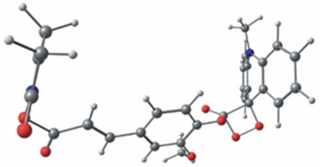	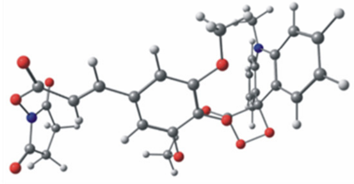	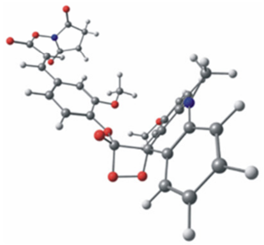

Entity no. (Scheme S4)	Compound (Scheme S3)		
	**AL4**	**AL5**	**AL6**
**a**	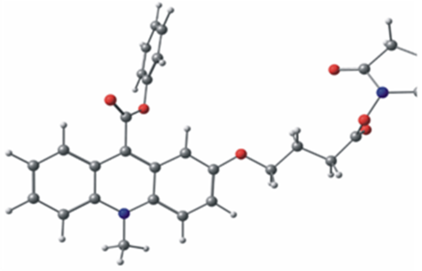	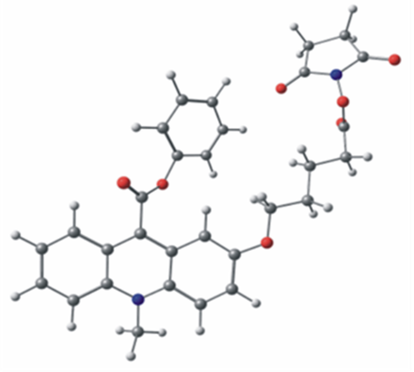	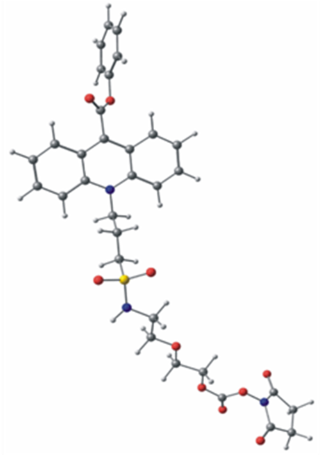
**b**	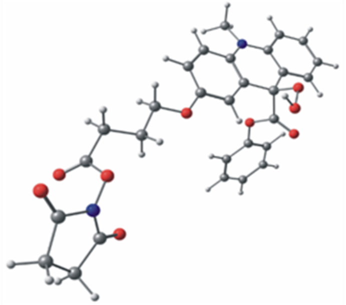	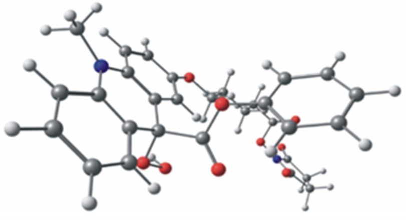	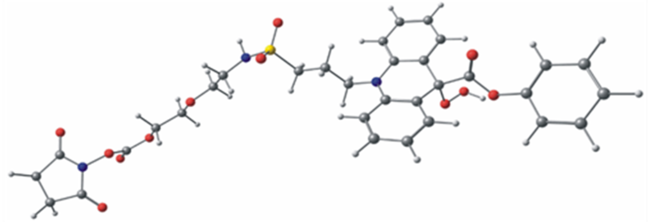
**c**	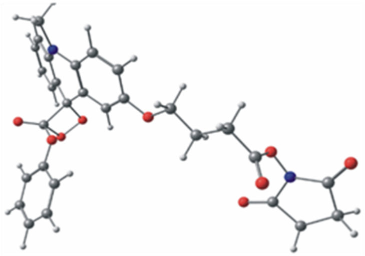	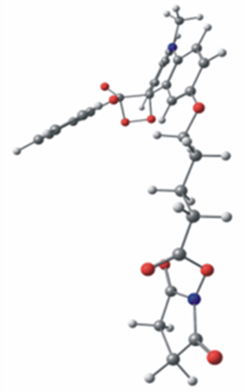	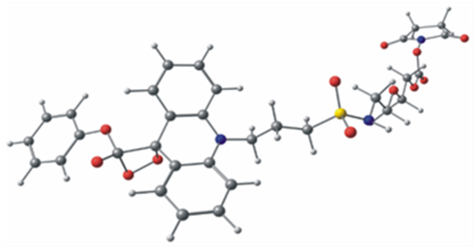

Entity no. (Scheme S4)	Compound (Scheme S3)			
	**C**	**AL1**	**AL2**	**AL3**
**d**		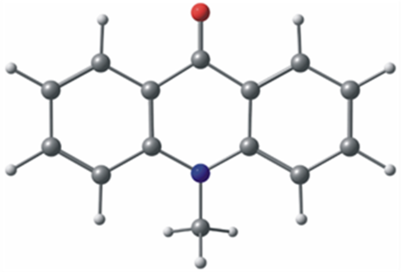		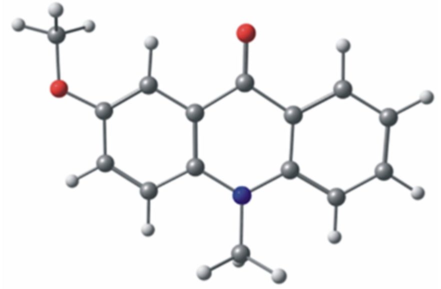
**f**	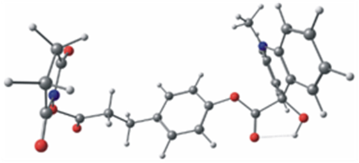	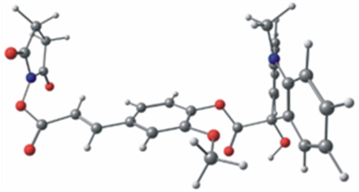	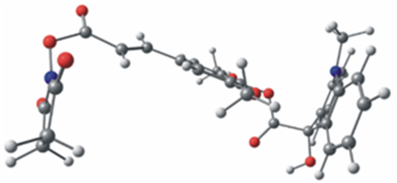	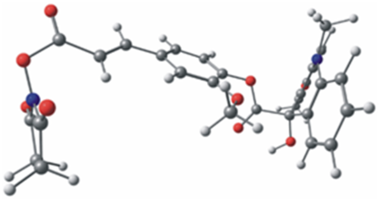
**g**	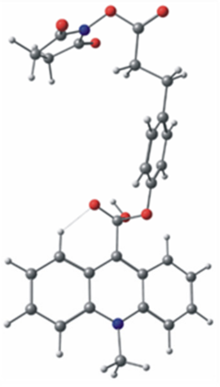	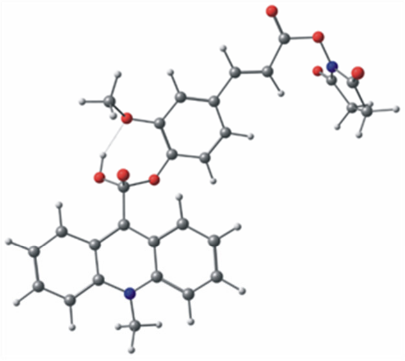	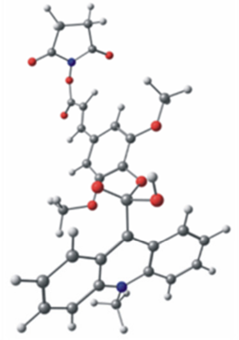	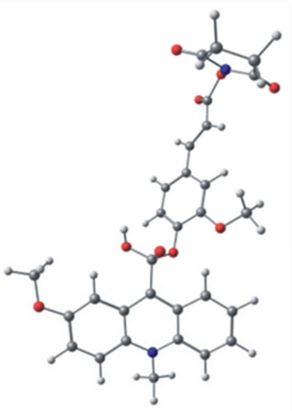

Entity no. (Scheme S4)	Compound (Scheme S3)		
	**AL4**	**AL5**	**AL6**
**d**	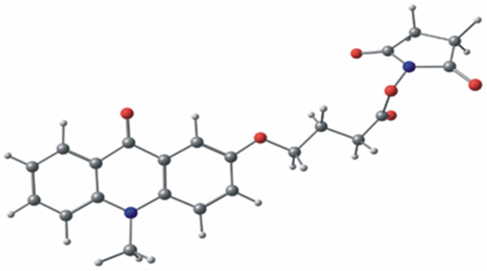	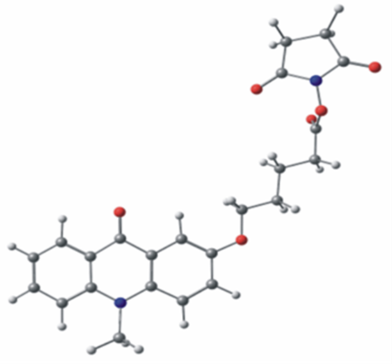	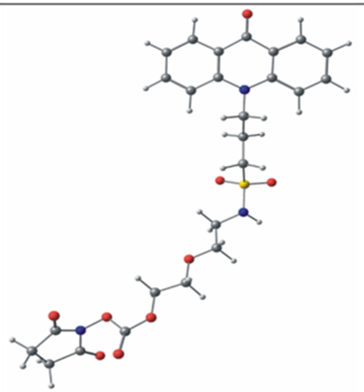
**f**	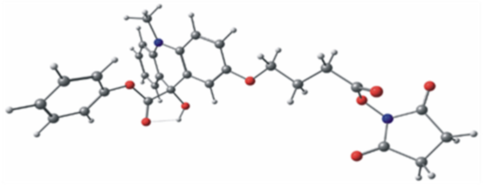	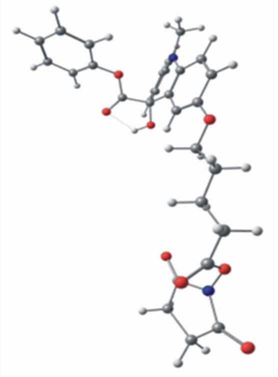	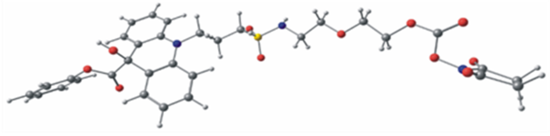
**g**	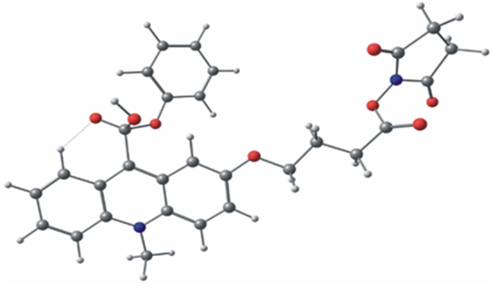	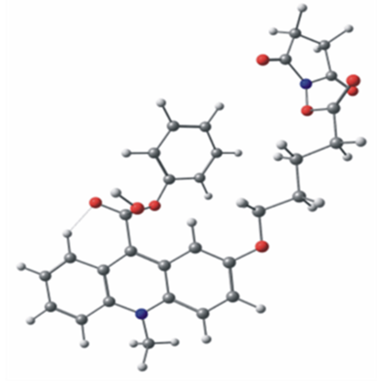	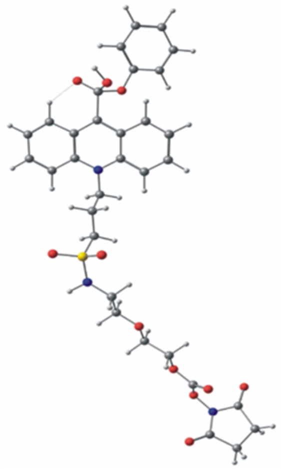

Entity no. (Scheme S4)	Compound (Scheme S3)			
	**C**	**AL1**	**AL2**	**AL3**
**h**		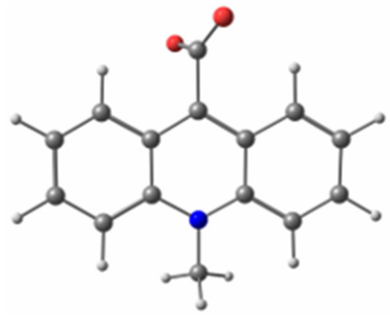		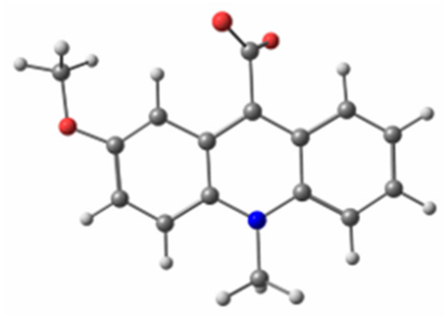
**TS1**	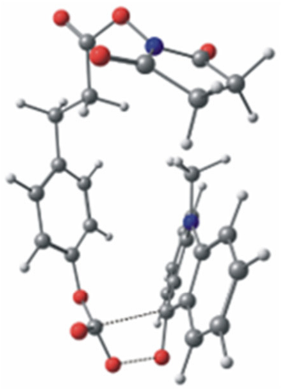	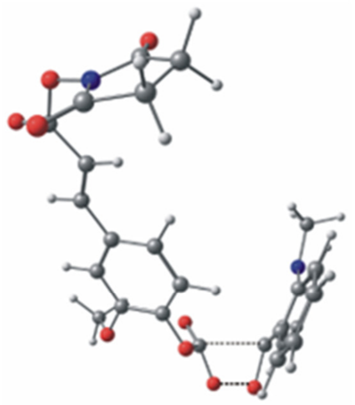	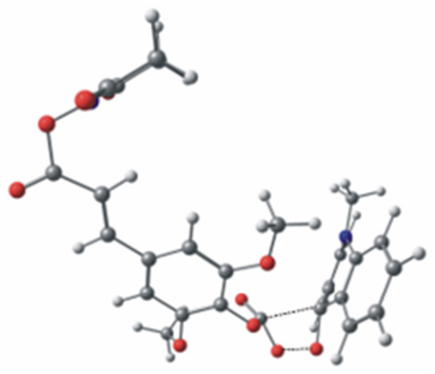	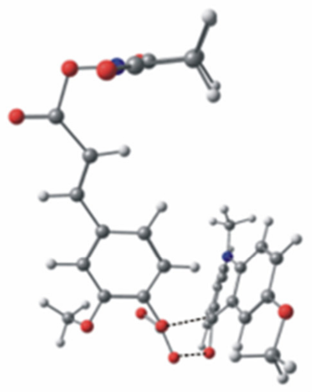

Entity no. (Scheme S4)	Compound (Scheme S3)		
	**AL4**	**AL5**	**AL6**
**h**	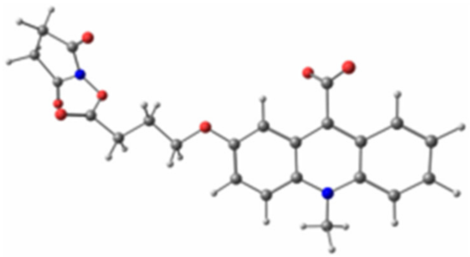	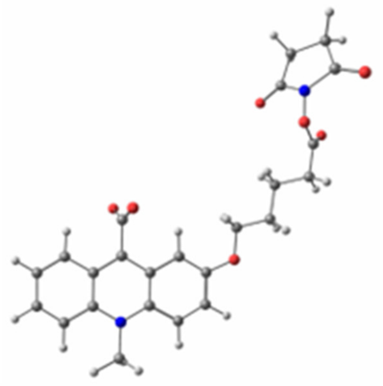	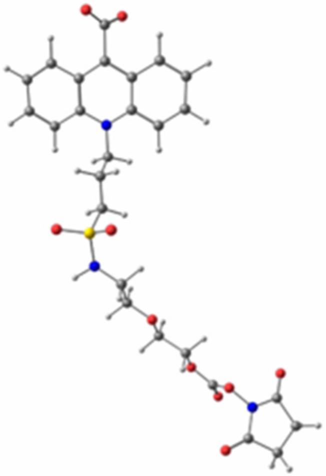
**TS1**	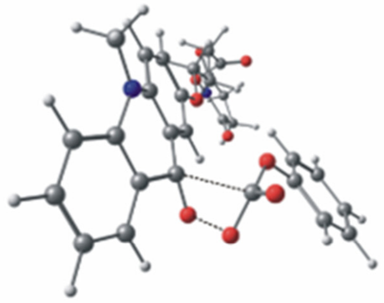	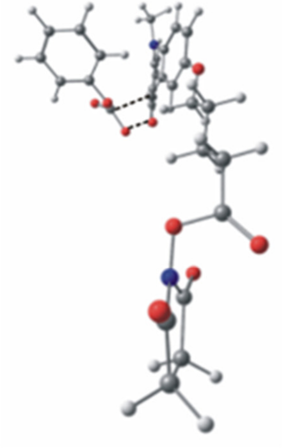	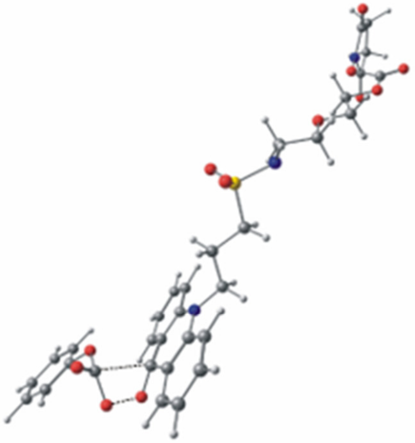

Figure S2. DFT (M06-2X/6-31G**) optimized geometries of selected entities occurring on the reaction pathways of AL's with OOH– and OH– (TS1 represent transition state geometries; the dotted lines denote the bonds broken during the course of the reaction).

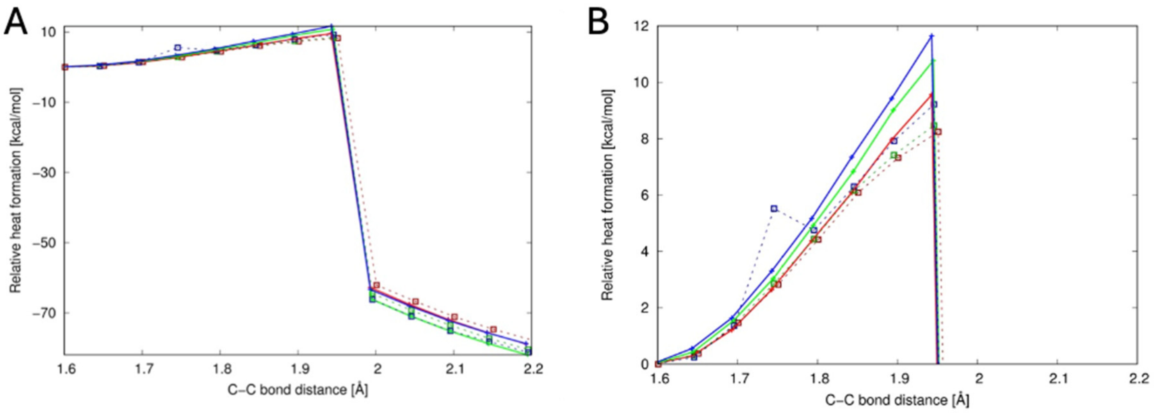

Figure S3. Influence of dielectric environment and cation proximity on the calculated reaction energetics of the acridinium chemiluminescence pathway. (A) Relative heat of formation along the reaction coordinate calculated for different dielectric constants (ε = 78.1, 10, 4) in the absence and presence of a nearby cation. (B) Comparison of calculated activation barriers for the chemiluminescence-generating step under different dielectric conditions with and without cation interaction.

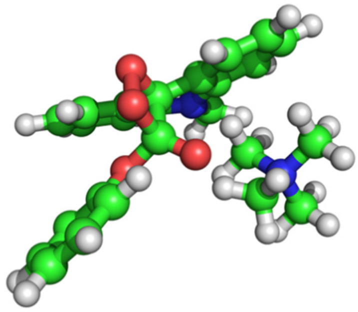

Figure S4. Optimized geometry of the acridan spiro intermediate in the presence of a nearby cation.

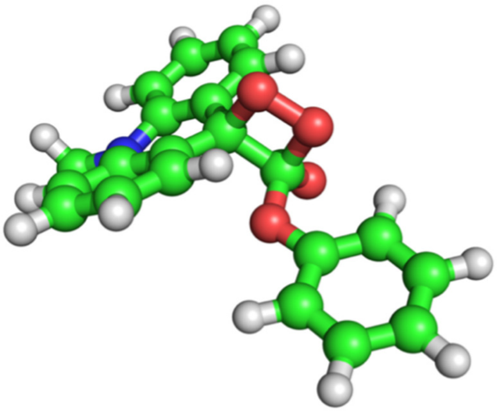

Figure S5. Structure of acridan spiroxy phenoxy alkoxide after PM7 geometry optimization in water.
